# High Working Capacity Acetylene Storage at Ambient Temperature Enabled by a Switching Adsorbent Layered Material

**DOI:** 10.1021/acsami.1c06241

**Published:** 2021-05-13

**Authors:** Shi-Qiang Wang, Xiao-Qing Meng, Matthias Vandichel, Shaza Darwish, Ze Chang, Xian-He Bu, Michael J. Zaworotko

**Affiliations:** † Bernal Institute, Department of Chemical Sciences, University of Limerick, Limerick V94 T9PX, Ireland; ‡ School of Materials Science and Engineering, Nankai University, Tianjin 300350, China

**Keywords:** acetylene storage, structural switching, stepped sorption isotherms, 2D coordination network, flexible metal−organic material

## Abstract

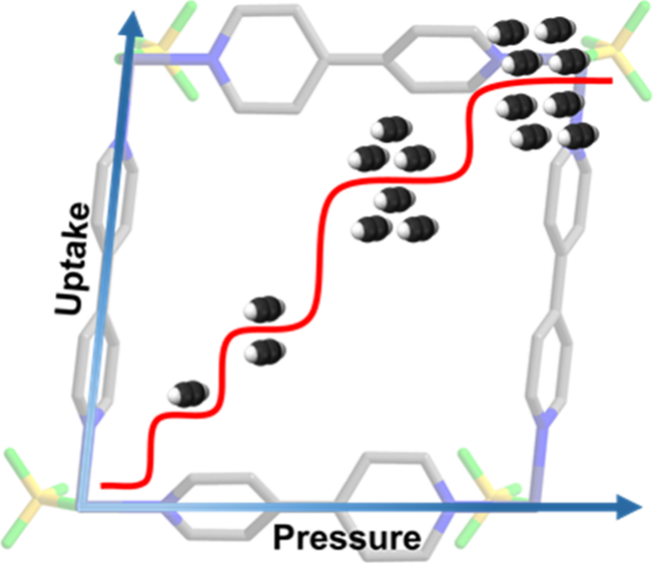

Unlike most gases, acetylene storage is a challenge because of its inherent pressure sensitivity. Herein, a square lattice (**sql**) coordination network [Cu(4,4′-bipyridine)_2_(BF_4_)_2_]_*n*_ (**sql-1-Cu-BF**_**4**_) is investigated with respect to its C_2_H_2_ sorption behavior from 189 to 298 K. The C_2_H_2_ sorption studies revealed that **sql-1-Cu-BF**_**4**_ exhibits multistep isotherms that are temperature-dependent and consistent with the transformation from “closed” (nonporous) to four “open” (porous) phases induced by the C_2_H_2_ uptake. The Clausius–Clapeyron equation was used to calculate the performance of **sql-1-Cu-BF**_**4**_ for C_2_H_2_ storage at pressures >1 bar, which revealed that its volumetric working capacity at 288 K is slightly superior to acetone (174 vs 170 cm^3^ cm^–3^) over a safer pressure range (1–3.5 vs 1–15 bar). Molecular simulations provided insights into the observed switching phenomena, revealing that the layer expansion of **sql-1-Cu-BF**_**4**_ occurs via intercalation and inclusion of C_2_H_2_. These results indicate that switching adsorbent layered materials offer promise for utility in the context of C_2_H_2_ storage and delivery.

## Introduction

Acetylene (C_2_H_2_) is an important feedstock for a range of chemical products including vinyl chloride, 1,4-butanediol, and acetylene black.^1^ Unfortunately, its high chemical reactivity can lead to explosive decomposition at moderate pressures ignited by a spark, shock wave, heat, or compression.^2^ The wide range of flammability (2.5–82% in air^3^) also makes the handling of C_2_H_2_ more challenging than most gases. Its flammable and explosive nature means that ca*.* 80% of C_2_H_2_ is used in situ without further shipping or storage,^4^ the balance being mainly used in oxyacetylene torches, for which C_2_H_2_ is stored in pressurized containers.

Attempts to store C_2_H_2_ directly under high pressure or liquefaction resulted in fatal detonations at the end of the 19th century.^3^ Current C_2_H_2_ storage technology involves desensitization by dissolving C_2_H_2_ in acetone that is predispersed in a porous monolith that completely fills a gas cylinder.^2,3^ The solubility of C_2_H_2_ in acetone at 15 °C is 29.3 cm^–3^ g^–1^ (22.5 cm^–3^ cm^–3^) at 1 bar, increasing to 470.4 cm^–3^ g^–1^ (192.3 cm^–3^ cm^–3^) at 15 bar (Tables S1 and S2, Figure S1). This large solubility difference means that ∼90% of the dissolved C_2_H_2_ can be delivered between 15 and 1 bar. However, discharging C_2_H_2_ is accompanied by the release of acetone vapor,^3^ preventing its use in the production of fine chemicals and electronic materials. Solid sorbents could address these safety matters as well as broaden the utility of pure C_2_H_2_ in this “age of gas”.^5^

Metal–organic materials (MOMs),^6,7^ especially metal–organic frameworks^8,9^ or porous coordination polymers,^10,11^ have attracted attention from academia and industry thanks to their properties and potential utility for storage, separation, and catalysis.^12−14^ An attribute of MOMs is their inherent modularity, which enables fine-tuning through crystal engineering or reticular chemistry.^15,16^ Early studies in the 1990s mainly focused on rigid MOMs, but there has been an increasing interest in flexible MOMs (FMOMs) since the 2000s, thanks to stimuli-responsive phenomena such as breathing, swelling, and gate-opening.^17−22^

With respect to C_2_H_2_ storage, over 100 MOMs have been investigated for C_2_H_2_ sorption (Table S3) since Kitagawa’s group reported that [Cu_2_(pzdc)_2_(pyz)] exhibits relatively low C_2_H_2_ uptake (42 cm^3^ g^–1^).^23^ Subsequent studies from Chen (UTSA series), Hong (FJI series), Xiang (FNU series), Qian (ZJU series), Bai (NJU-Bai series), He (ZJNU series), Schröder (MFM series), and others have set new C_2_H_2_ uptake benchmarks.^24−35^ However, such studies mainly focused on the C_2_H_2_ uptake, whereas the key performance metric is the working capacity under practically relevant conditions.^36,37^ Although there is no target for the working pressure range between delivery pressure (*P*_de_) and storage pressure (*P*_st_), a residual pressure for C_2_H_2_ of 1.0 bar (i.e., gauge pressure 0 bar) is the most practically relevant value of *P*_de_.^26,30^ Otherwise, the discharging rate will be low and air could backfill a cylinder. Regarding *P*_st_, 2.0 bar is generally regarded as the safe limit for pure C_2_H_2_.^38^ In the case of acetone, the formation of acetone–C_2_H_2_ clathrates desensitizes C_2_H_2_,^39^ allowing it to be safely stored in acetone up to 18 bar at 21 °C (equivalent to ca*.* 15 bar at 15 °C).^2^ C_2_H_2_ molecules isolated in the pores of MOMs could also suppress the explosive nature of C_2_H_2_. With this in mind, 1–15 bar is a realistic working pressure range for C_2_H_2_ storage/delivery using physisorbents.

**Scheme 1:**
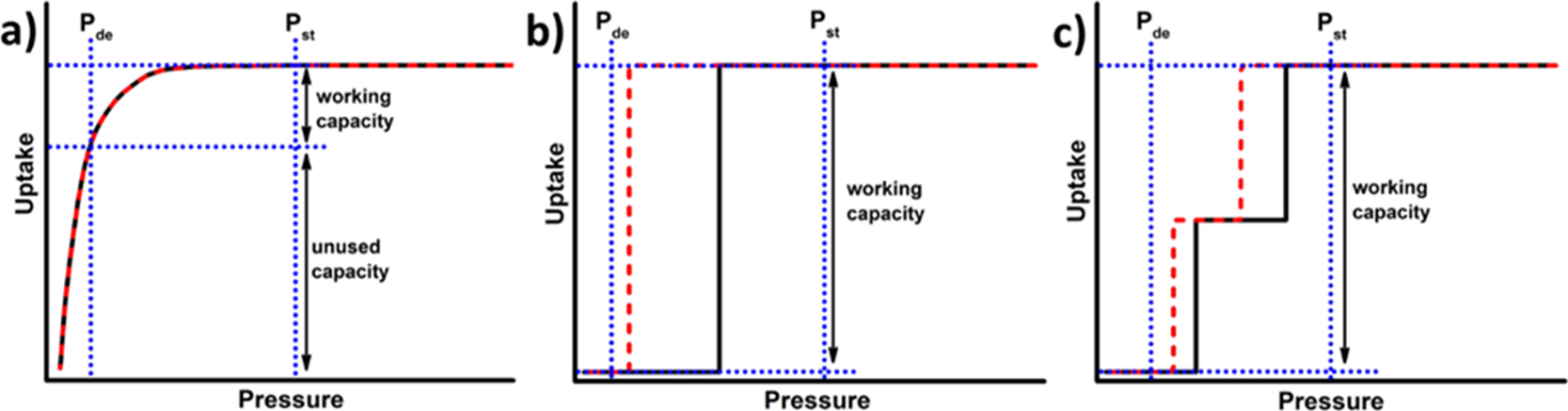
Three Types of Gas Sorption Isotherms (a) Type I; (b) type F-IV (steps = 1); and (c) type F-IV^*m*^ (steps ≥2). Black solid line: adsorption; red dashed line: desorption; *P*_de_: delivery pressure; and *P*_st_: storage pressure.

In this context, the fact that most physisorbents exhibit type I isotherms (Scheme 1a) means that the working capacity is usually less than the uptake capacity since the adsorbate remains present at *P*_de_. Conversely, when a sorbent exhibits a type F-IV^40^ or stepped isotherm (Scheme 1b), the working capacity can match the uptake capacity. These desired isotherms can also comprise multiple steps^41,42^ (i.e., type F-IV^*m*^, Scheme 1c). If there is hysteresis, it requires that *P*_de_ must be lower than the gate desorption pressure (*P*_gd_) rather than the gate adsorption pressure (*P*_ga_).

C_2_H_2_ sorption studies are typically reported from 0 to 1 bar for safety reasons, precluding direct measure of the storage performance at elevated pressures (e.g., 1–15 bar). Indeed, the benchmark MOMs for the C_2_H_2_ uptake at 1 bar are disadvantageous for C_2_H_2_ delivery because they tend to retain most of the sorbed C_2_H_2_ even at 0.5 bar (Figure S2). In effect, this means that rigid MOMs with high uptake capacity at 1 bar are suited for C_2_H_2_ sequestration rather than C_2_H_2_ storage/delivery. For FMOMs that transform from nonporous to porous phases, the situation is different. Furthermore, *P*_ga_ and *P*_gd_ can be calculated by applying the Clausius–Clapeyron equation,^43−45^ providing an indirect approach to determine the C_2_H_2_ working capacity at higher pressures and avoiding the explosion risks.

We have studied the sorption behavior of the square lattice (**sql**) coordination network [Co(bpy)_2_(NCS)_2_]_*n*_ (bpy = 4,4′-bipyridine), **sql-1-Co-NCS**, and found that it exhibits a reversible switching behavior induced by CO_2_ or C_8_ aromatic isomers.^46,47^ Although **sql-1-Co-NCS** is a type of FMOM, its sudden switching behavior makes it distinctive^48^ and more desirable than FMOMs with type F-I or F-II isotherms.^49,50^ We classified this type of 2D MOM as a switching adsorbent layered material (SALMA).^47^ The prototypal SALMA is the related **sql** coordination network [Cu(bpy)_2_(BF_4_)_2_]_*n*_, also known as **ELM-11**.^51^ The switching behavior of **ELM-11** has been studied for a range of adsorbates such as CO_2_, N_2_, CH_4_, O_2_, Ar, and *n*-butane.^51−54^ The storage potential for fuel gases such as CH_4_ has also been investigated for **ELM-11**, a type F-IV isotherm being observed with a working capacity the same as the uptake capacity (80 cm^3^ g^–1^).^52,53^ A more recent study investigated the C_2_H_2_ sorption properties of **ELM-11** under ambient conditions but mainly focused on C_2_H_2_/C_2_H_4_ separation.^55^**ELM-11** and **sql-1-Co-NCS** belong to the same family with the general formula [**M**(**L**)_2_(**A**)_2_]_*n*_.^56^ Herein, we report on **ELM-11**, which we refer to as **sql-1-Cu-BF**_**4**_, in terms of its C_2_H_2_ sorption from 189 to 298 K and discuss the implications of these results for C_2_H_2_ storage.

## Results and Discussion

**Figure 1:**
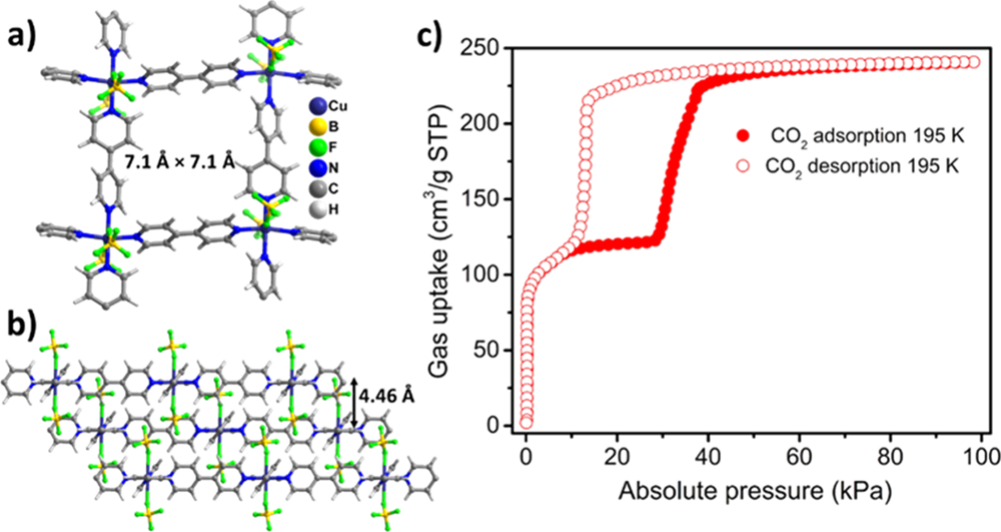
The crystal structure of **sql-1-Cu-BF**_**4**_ reveals (a) a square cavity with an effective size of 7.1 Å × 7.1 Å and an (b) interlayer distance of 4.46 Å. (c) CO_2_ sorption for **sql-1-Cu-BF**_**4**_ at 195 K.

**sql-1-Cu-BF**_**4**_ in its nonporous form was prepared by heating its precursor {[Cu(bpy)(BF_4_)_2_(H_2_O)_2_]·(bpy)}_*n*_ (purchased from TCI) at 373 K under vacuum for 5 h. The effective cavity size of **sql-1-Cu-BF**_**4**_ is 7.1 Å × 7.1 Å (Figure 1a) and the interlayer distance is 4.46 Å (Figure 1b), both comparable to those of **sql-1-Co-NCS**.^46^ We collected CO_2_ sorption isotherms at 195 K for **sql-1-Cu-BF**_**4**_ (Figure 1c). Both the profiles of the two-step isotherm and the measured saturation uptake (245 cm^3^ g^–1^) are consistent with previously reported parameters.^45,57^

**Figure 2:**
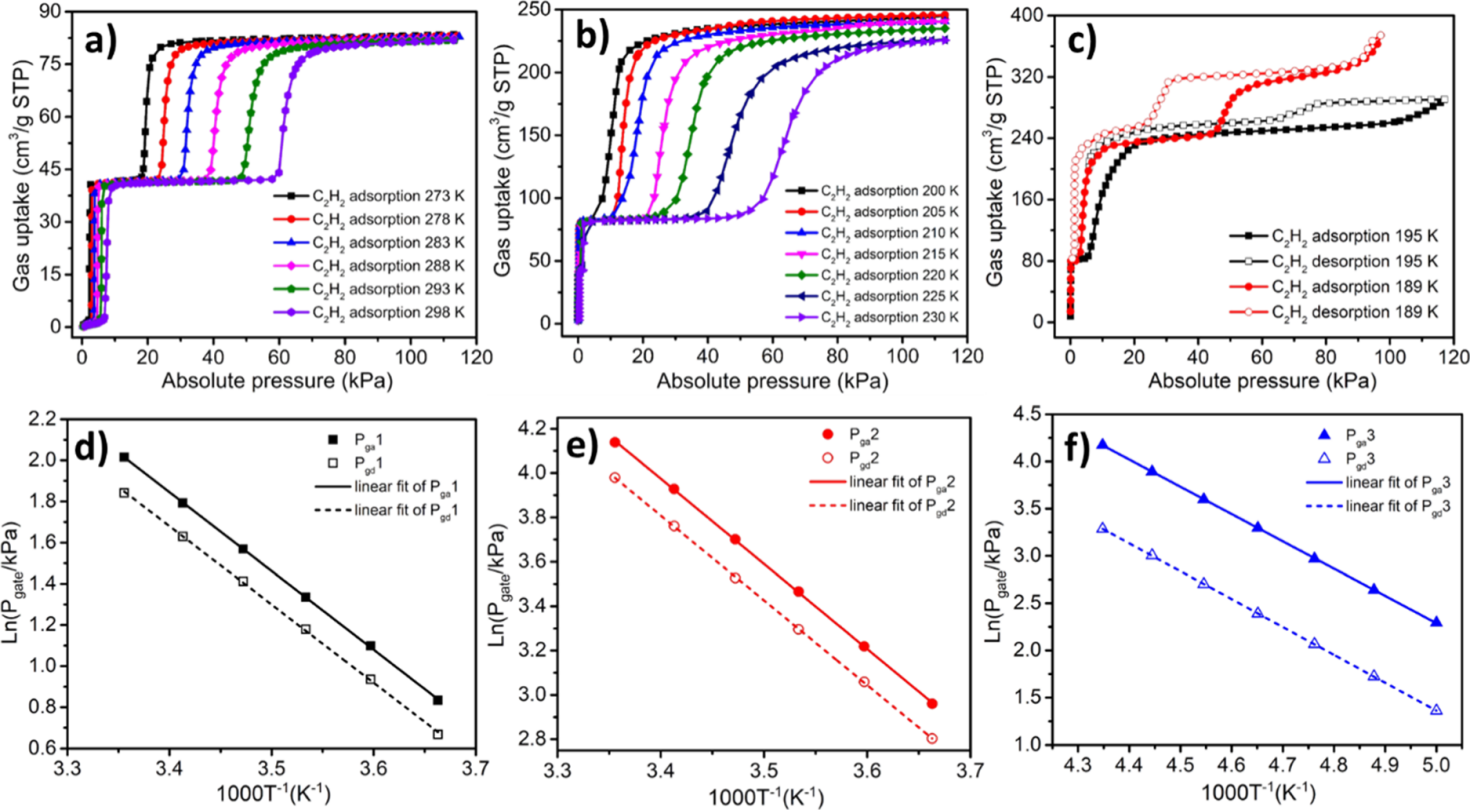
C_2_H_2_ sorption isotherms of **sql-1-Cu-BF**_**4**_: (a) 273–298; (b) 200–230; and (c) 189–195 K. Linear fits of gate sorption pressure (LnP) and temperature (1000/*T*) using the Clausius–Clapeyron equation for (d–f) the first, second, and third gate adsorption (*P*_ga_1/*P*_ga_2/*P*_ga_3) and desorption pressures (*P*_gd_1/*P*_gd_2/*P*_gd_3), respectively.

C_2_H_2_ sorption isotherms for **sql-1-Cu-BF**_**4**_ at 273 and 298 K have been reported;^55^ we recollected the C_2_H_2_ sorption isotherms at 5 K intervals with more data points (Figures 2a and S3a). The isotherms measured up to 115 kPa reveal two steps. The uptake was found to be 41 cm^3^ g^–1^ at the first step and 82 cm^3^ g^–1^ at the second step, consistent with the previous report^55^ and equivalent to one and two C_2_H_2_ molecules per formula unit, respectively (denoted herein as **sql-1-Cu-BF**_**4**_**·1C**_**2**_**H**_**2**_ and **sql-1-Cu-BF**_**4**_**·2C**_**2**_**H**_**2**_). The first and second gate adsorption pressures (*P*_ga_1 and *P*_ga_2) at 273 K are 2.3 and 19.3 kPa, respectively, both lower than *P*_ga_1 of CO_2_ (ca*.* 35 kPa at 273 K).^51−53^ We ascribe these differences to stronger sorbate–sorbent interactions with C_2_H_2_ due to its higher polarizability.^13^

CO_2_ sorption studies on **sql-1-Cu-BF**_**4**_ revealed that there is an additional higher uptake step at around 30 kPa when measured at 195 K (Figure 1c).^45,57^ We therefore wondered if C_2_H_2_ might also exhibit higher uptake steps at a lower temperature. Indeed, when C_2_H_2_ isotherms were collected between 200 and 230 K (Figures 2b and S3b), a third step with ca*.* 245 cm^3^ g^–1^ uptake was observed. This step matches the CO_2_ uptake at 195 K above 30 kPa and is equivalent to six C_2_H_2_ molecules per formula unit (denoted as **sql-1-Cu-BF**_**4**_**·6C**_**2**_**H**_**2**_). Surprisingly, at 195 K, a fourth step appeared at around 100 kPa (Figure 2c). Further cooling to 189 K revealed that this new phase offered an uptake capacity of ca*.* 325 cm^3^ g^–1^, equivalent to eight C_2_H_2_ molecules per formula unit (denoted as **sql-1-Cu-BF**_**4**_**·8C**_**2**_**H**_**2**_).

**Figure 3:**
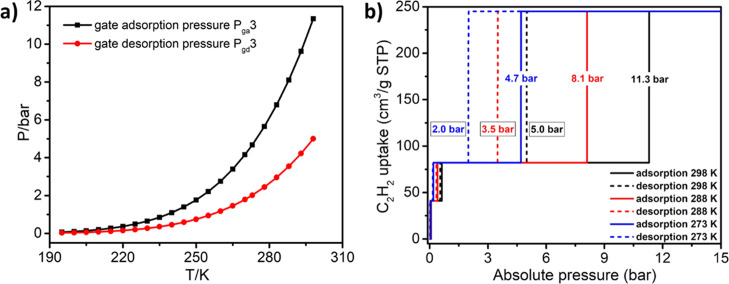
(a) Calculated plots of *P*_ga_3 (*P*_gd_3) from 195 to 298 K and (b) ideal high-pressure C_2_H_2_ sorption isotherms for **sql-1-Cu-BF**_**4**_ at 273, 288, and 298 K.

We correlated the gate sorption pressure and temperature by applying the Clausius–Clapeyron equation d ln *P*/(d(1/*T*)) = −Δ*H*/*R* (Figures 2d–f, S4 and Table S4).^43−46^ The formation/dissociation enthalpies Δ_f_*H*/Δ_d_*H* of the first three steps were determined to be 31.8/31.6, 31.9/31.8, and 24.0/24.5 kJ mol^–1^ for the first, second, and third adsorption/desorption steps, respectively. The calculated plots of *P*_ga_3 (*P*_gd_3) from 195 to 298 K are given in Figure 3a. The extrapolated values of *P*_ga_3 (*P*_gd_3) are 4.7 (2.0), 8.1 (3.5), and 11.3 (5.0) bar at 273, 288, and 298 K, respectively. Ideal C_2_H_2_ sorption isotherms (up to 15 bar) at these three temperatures are plotted in Figure 3b and are based on the experimental (<1.0 bar) and calculated (>1.0 bar) gate sorption pressures.

**Figure 4:**
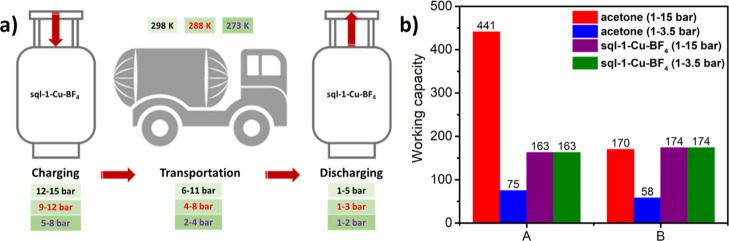
(a) Proposed charging, transportation, and discharging pressures of C_2_H_2_ at 298, 288, and 273 K; (b) C_2_H_2_ working capacity of acetone and **sql-1-Cu-BF**_**4**_ at 288 K: A = gravimetric (cm^–3^ g^–1^); B = volumetric (cm^–3^ cm^–3^).

Compared with benchmark MOMs, which exhibit 200–250 cm^3^ g^–1^ C_2_H_2_ uptake under ambient conditions (Figure S2), **sql-1-Cu-BF**_**4**_ has a comparable or superior uptake (245 cm^3^ g^–1^ at the third plateau) at high pressure (e.g., >3.5 bar at 288 K). This suggests that **sql-1-Cu-BF**_**4**_ might be useful for practical C_2_H_2_ storage and delivery between 1 and 15 bar. Furthermore, the hysteresis gaps were calculated to be between 2.7 and 6.3 bar from 273 to 298 K, meaning that storage/transportation and discharge pressures could be lower than the charging pressure (Figure 4a).^32,58^ Although acetone has a higher gravimetric uptake and working capacity from 1 to 15 bar (Figures S5 and S6), **sql-1-Cu-BF**_**4**_ exhibits a much higher gravimetric working capacity between 1 and 3.5 bar (Figure 4b, group A). Moreover, the volumetric working capacity is an important parameter since the container volume is necessarily limited (Figure S7). Acetone has a relatively low density (0.785 g cm^–1^) and expands almost 100% when C_2_H_2_ is charged at 15 bar and 288 K. Its volumetric working capacity is therefore lower than its gravimetric value. Conversely, the network density of **sql-1-Cu-BF**_**4**_**·6C**_**2**_**H**_**2**_ excluding C_2_H_2_ is 1.07 g cm^–1^ (Table S5), meaning that its volumetric working capacity is higher than its gravimetric value. Overall, **sql-1-Cu-BF**_**4**_ has a slightly better volumetric working capacity (174 *vs* 170 cm^–3^ cm^–3^) than acetone but in a lower and therefore safer working pressure range (Figure 4b, group B).

**Figure 5:**
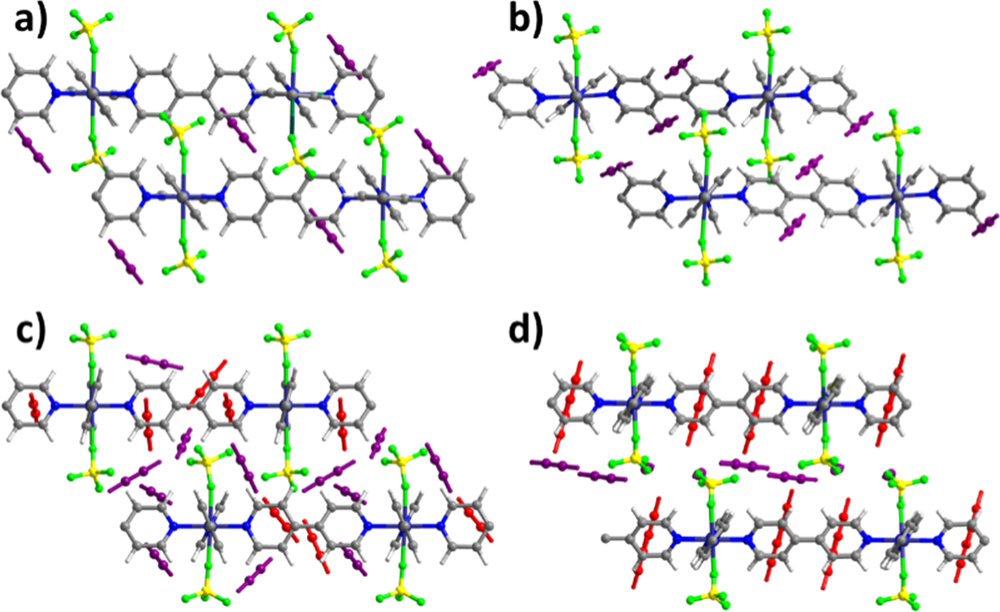
Calculated C_2_H_2_ sites in (a) **sql-1-Cu-BF**_**4**_**·1C**_**2**_**H**_**2**_; (b) **sql-1-Cu-BF**_**4**_**·2C**_**2**_**H**_**2**_; (c) **sql-1-Cu-BF**_**4**_**·6C**_**2**_**H**_**2**_; and (d) **sql-1-Cu-BF**_**4**_**·8C**_**2**_**H**_**2**_.

To better understand stepwise the C_2_H_2_ isotherms exhibited by **sql-1-Cu-BF**_**4**_, molecular simulations using the density functional theory (DFT) were conducted based on structural analogues.^44,47,57^ As revealed by Figure 5, C_2_H_2_ molecules (violet) occupy the interlayer space in **sql-1-Cu-BF**_**4**_**·1C**_**2**_**H**_**2**_ (Figure 5a) and **sql-1-Cu-BF**_**4**_**·2C**_**2**_**H**_**2**_ (Figure 5b). Extra C_2_H_2_ molecules (red) reside in the square cavity for **sql-1-Cu-BF**_**4**_**·6C**_**2**_**H**_**2**_ (Figure 5c) and **sql-1-Cu-BF**_**4**_**·8C**_**2**_**H**_**2**_ (Figure 5d). This combination of intercalation and inclusion is stoichiometric as experimentally observed for xylenes in **sql-1-Co-NCS**.^47^

Analysis of C_2_H_2_ binding sites in these four phases (Figures S8–S10) reveals adsorbate–adsorbent (i.e., C–H···F and C≡C···π) and adsorbate–adsorbate (i.e., C–H···C≡C) interactions. The shortest C–H···F hydrogen-bond distances in **sql-1-Cu-BF**_**4**_**·*x*C**_**2**_**H**_**2**_ (***x*** = 1, 2, 6, and 8), 3.381, 2.666, 2.069, and 2.068 Å, respectively, match adsorbent–C_2_H_2_ contacts (1.980–3.938 Å) in SIFSIX sorbents.^59^ In addition, van der Waals C_2_H_2_–C_2_H_2_ and C_2_H_2_–bpy interactions were found in **sql-1-Cu-BF**_**4**_**·*x*C**_**2**_**H**_**2**_ (***x*** = 6, 8) (Figures S9 and S10). The interlayer distance of each phase is 5.74, 5.79, 6.97, and 7.96 Å, respectively, meaning 28.7, 29.8, 56.3, and 78.5% increase compared with that of the closed phase (4.46 Å). The expansion of adjacent layers is endothermic, while contraction is exothermic, suppressing heat release during adsorption and the endothermic nature of desorption. Such intrinsic heat management is desirable in sorbents and could make the charging process both safer and faster.^57,60^

## Conclusions

In conclusion, **sql-1-Cu-BF**_**4**_ offers several advantageous features for C_2_H_2_ storage: (a) commercial availability of its precursor; (b) good recyclability and fast sorption kinetics;^52,53,61^ (c) operating pressures compatible with acetone-related technology; and (d) release of pure C_2_H_2_ as there are no other volatiles present. A drawback is its hydrophilicity,^62^ which could cause a competition with the water vapor and decrease the lifetime of the sorbent.^63^ It is reasonable to assume that the inclusion and intercalation of C_2_H_2_ into **sql-1-Cu-BF**_**4**_ will desensitize C_2_H_2_ enough to enable the loading of C_2_H_2_ from 1 to 15 bar without the risk of explosion. This matter must be verified or a porous monolith might be needed. Nevertheless, **sql-1-Cu-BF**_**4**_ offers C_2_H_2_ storage under mild operating pressures and its modularity suggests that a platform of related materials will be accessible.

## Experimental Methods

### Gas Sorption Experiments

Gas sorption experiments of **sql-1-Cu-BF**_**4**_ were conducted on a Micromeritics 3flex instrument (195 K CO_2_ and 189, 195, and 273–298 K C_2_H_2_) and an ASAP 2020 instrument (200–230 K C_2_H_2_). The precursor {[Cu(bpy)(BF_4_)_2_(H_2_O)_2_]·(bpy)}_*n*_ (purchased from TCI, CAS no. 854623-98-6) was activated under vacuum at 100 °C for 5 h to transform to **sql-1-Cu-BF**_**4**_ by using a Smart VacPrep instrument prior to the analysis. 195 K CO_2_ and C_2_H_2_ experiments were maintained by a 4 L Dewar flask filled with the mixture of acetone and dry ice. 189 K C_2_H_2_ experiments were maintained by a 4 L Dewar flask filled with the mixture of ethyl acetate and liquid nitrogen with care (part of ethyl acetate was solidified, accompanied with white fog). 273–298 K C_2_H_2_ experiments were maintained by a Julabo F25-ME Chiller, while 200–230 K C_2_H_2_ experiments were maintained by a LAUDA PRO RP890 Chiller.

### DFT Calculations

All periodic DFT calculations were carried out using Castep as implemented in the Materials Studio package. Vanderbilt-type ultrasoft pseudopotentials and the generalized gradient approximation with the Perdew–Burke–Ernzerhof exchange correlation were used for all structure calculations. A semiempirical dispersion correction was included in the calculation to take into account the van der Waals interactions. A cutoff energy of 544 eV and a 3 × 3 × 2 *k*-point mesh were found to be sufficient for the total energy to converge within 0.05 meV/atom. The starting structures were obtained from the literature (see Table S4). The CO_2_ molecules in the structures of **sql-1-Cu-BF**_**4**_**·2CO**_**2**_ and **sql-1-Cu-BF**_**4**_**·6CO**_**2**_ were replaced by C_2_H_2_ molecules to form the initial structures of **sql-1-Cu-BF**_**4**_**·2C**_**2**_**H**_**2**_ and **sql-1-Cu-BF**_**4**_**·6C**_**2**_**H**_**2**_, and then, the location of C_2_H_2_ molecules was optimized using the abovementioned method. For **sql-1-Cu-BF**_**4**_**·C**_**2**_**H**_**2**_, only half of the C_2_H_2_ molecules in the structure of **sql-1-Cu-BF**_**4**_**·2C**_**2**_**H**_**2**_ were kept and located in the symmetric position. Similarly, for **sql-1-Cu-BF**_**4**_**·8C**_**2**_**H**_**2**_, the structures of **sql-1-Cu-BF**_**4**_**·6C**_**2**_**H**_**2**_ and **sql-1-Co-NCS·4PX** were referred. Half of the C_2_H_2_ molecules were located in the square cavity and the other half were located in the interlayer space. The coordination network was set as flexible to allow the layer contraction/expansion. All the simulations on the structures were conducted in the *P*1 space group.

## Supporting Information Available

C_2_H_2_ desorption isotherms, performance evaluations of rigid benchmark MOMs, acetone and **sql-1-Cu-BF**_**4**_, and Clausius–Clapeyron calculations (PDF)
Simulated structural file of **sql-1-Cu-BF**_**4**_**·C**_**2**_**H**_**2**_ (CIF)
Simulated structural file of **sql-1-Cu-BF**_**4**_**·2C**_**2**_**H**_**2**_ (CIF)
Simulated structural file of **sql-1-Cu-BF**_**4**_**·6C**_**2**_**H**_**2**_ (CIF)
Simulated structural file of **sql-1-Cu-BF**_**4**_**·8C**_**2**_**H**_**2**_ (CIF)

## Supplementary Material

am1c06241_si_001.pdf

am1c06241_si_002.cif

am1c06241_si_003.cif

am1c06241_si_004.cif

am1c06241_si_005.cif
