# Supplementary material for: High Working Capacity Acetylene Storage at Ambient Temperature Enabled by a Switching Adsorbent Layered Material
Source: ACS Appl Mater Interfaces. 2021 May 13;13(20):23877–83. doi: 10.1021/acsami.1c06241 (PMC8289182; doi:10.1021/acsami.1c06241)
Supplement: Supplementary file 1 — am1c06241_si_001.pdf [file am1c06241_si_001.pdf]

Supporting Information

High working capacity acetylene storage at ambient temperature enabled by a switching adsorbent layered material

Shi-Qiang Wang[1], Xiao-Qing Meng[2], Matthias Vandichel[1], Shaza Darwish[1], Ze Chang[2], Xian-He Bu[2], Michael J. Zaworotko[1]*

[1] Bernal Institute and Department of Chemical Sciences, University of Limerick, Limerick V94 T9PX, Republic of Ireland.

[2] School of Materials Science and Engineering, Nankai University, Tianjin 300350, China.

**E-mail:* Michael.Zaworotko@ul.ie

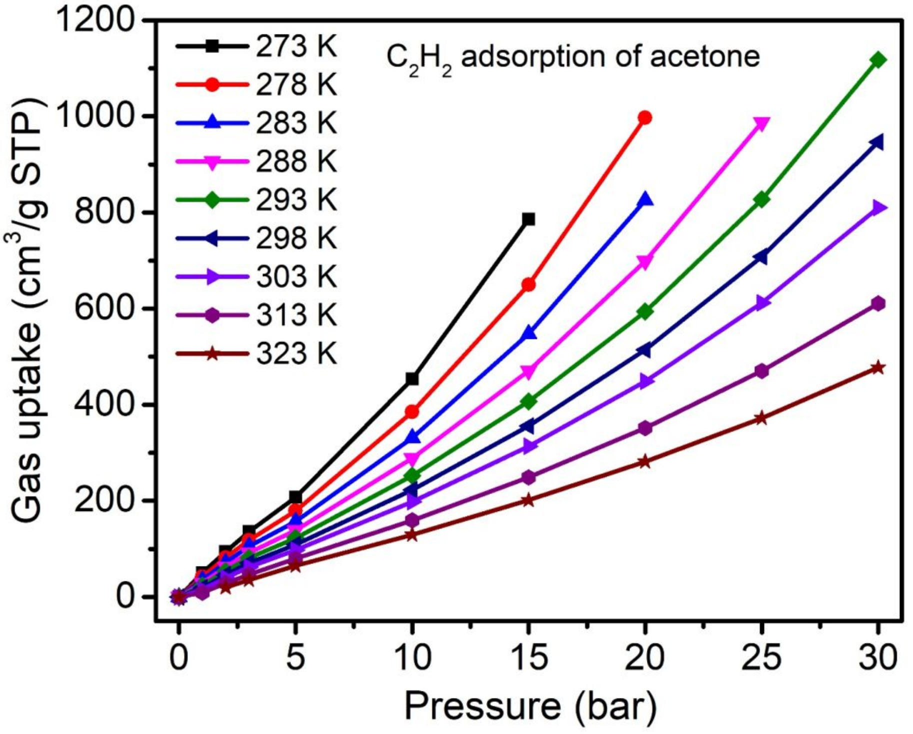

Figure S1. C_2H_2 adsorption isotherms of acetone derived from Table S1.

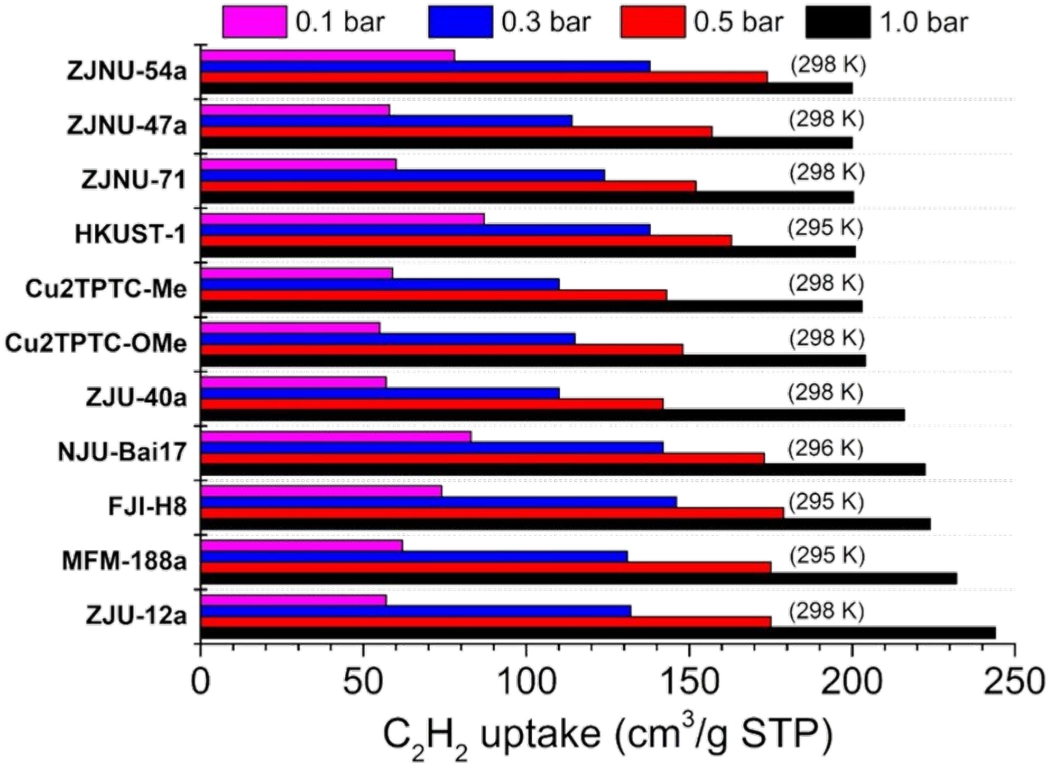

Figure S2. Comparison of current benchmark materials for C_2H_2 sorption at 0.1, 0.3, 0.5 and 1 bar. Between 0.5-1 bar, the working capacities of such sorbents are in the range of 26-74 cm^3/g, only accounting for 13-34% of their full uptakes.

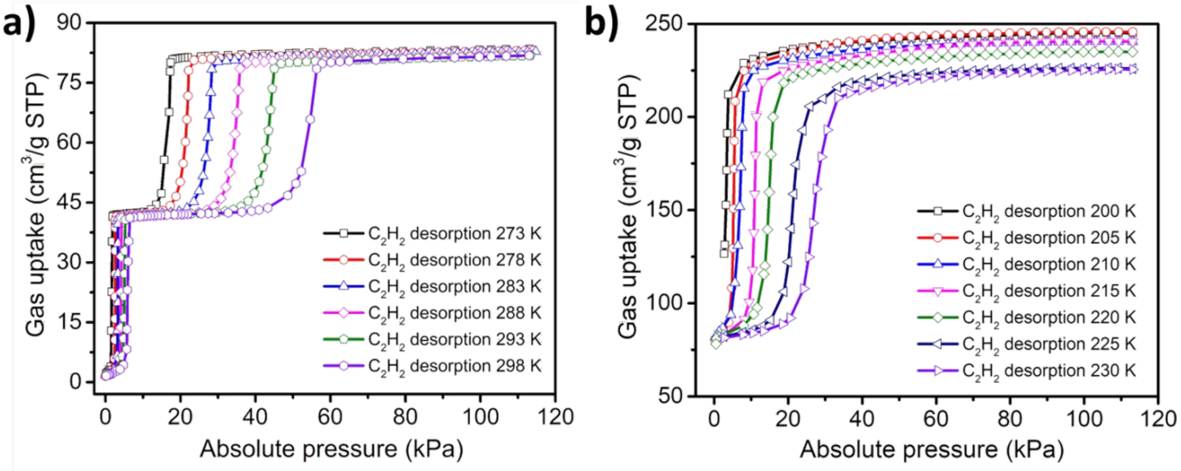

Figure S3. C_2H_2 desorption isotherms of **sql-1-Cu-BF$_4$** at (a) 273-298 K and (b) 200-230 K.

a)

T/K	$P_{ga}1$/kPa	$P_{gd}1$/kPa	$P_{ga}2$/kPa	$P_{gd}2$/kPa
273	2.3	1.95	19.3	16.5
278	3.0	2.55	25.0	21.3
283	3.8	3.25	32.0	27.0
288	4.8	4.1	40.5	34.0
293	6.0	5.1	50.8	43.0
298	7.5	6.3	62.7	53.5

T/K	$P_{ga}3$/kPa	$P_{gd}3$/kPa
200	9.9	3.9
205	14.0	5.6
210	19.5	7.9
215	27.0	10.9
220	36.5	14.9
225	49.0	20.2
230	65.0	26.7

b)

	$P_{ga}1$	$P_{gd}1$
Equation	y = a + b*x	y = a + b*x
Weight	No Weighting	No Weighting
Intercept	14.84821 ± 0.07127	14.6119 ± 0.121
Slope	-3.82453 ± 0.02032	-3.80364 ± 0.0345
Residual Sum of Squares	1.09179E-4	3.14699E-4
Pearson's r	-0.99994	-0.99984
R-Square(COD)	0.99989	0.99967
Adj. R-Square	0.99986	0.99959

	$P_{ga}2$	$P_{gd}2$
Equation	y = a + b*x	y = a + b*x
Weight	No Weighting	No Weighting
Intercept	17.02902 ± 0.06963	16.80373 ± 0.05442
Slope	-3.83948 ± 0.01985	-3.82214 ± 0.01552
Residual Sum of Squares	1.04217E-4	6.36533E-5
Pearson's r	-0.99995	-0.99997
R-Square(COD)	0.99989	0.99993
Adj. R-Square	0.99987	0.99992

	$P_{ga}3$	$P_{gd}3$
Equation	y = a + b*x	y = a + b*x
Weight	No Weighting	No Weighting
Intercept	16.72227 ± 0.02998	16.11686 ± 0.02281
Slope	-2.88685 ± 0.00643	-2.95098 ± 0.00489
Residual Sum of Squares	6.82546E-5	3.95059E-5
Pearson's r	-0.99999	-0.99999
R-Square(COD)	0.99998	0.99999
Adj. R-Square	0.99997	0.99998

$\ln(P_{ga}1) = -3825/T + 14.848$	$\ln(P_{gd}1) = -3804/T + 14.612$
$\ln(P_{ga}2) = -3839/T + 17.029$	$\ln(P_{gd}2) = -3822/T + 16.804$
$\ln(P_{ga}3) = -2887/T + 16.722$	$\ln(P_{gd}3) = -2951/T + 16.117$

Figure S4. (a) The linear fit parameters and (b) results by using Clausius-Clapeyron equation.

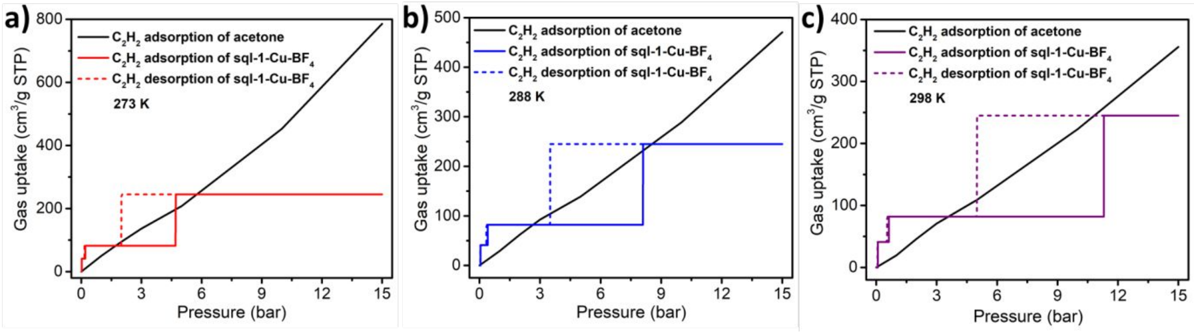

Figure S5. Comparison of C_2H_2 uptake between **sql-1-Cu-BF$_4$** and acetone at a) 273, b) 288, and c) 298 K.

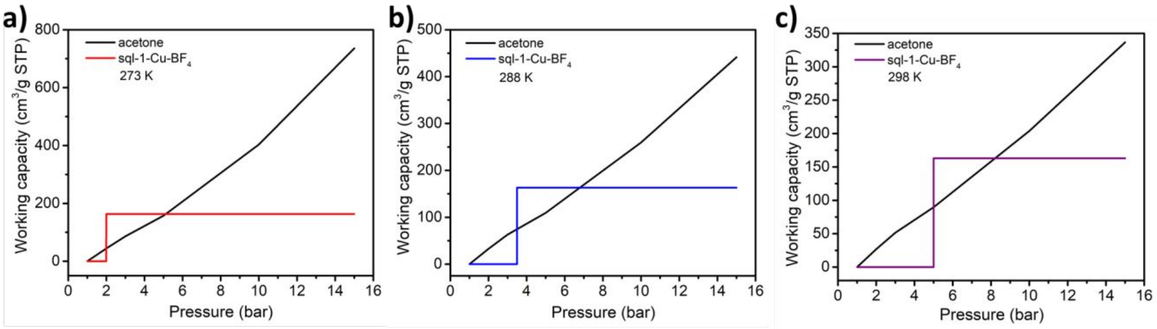

Figure S6. Comparison of gravimetric working capacity of C_2H_2 (1-15 bar) between **sql-1-Cu-BF$_4$** and acetone at a) 273, b) 288, and c) 298 K.

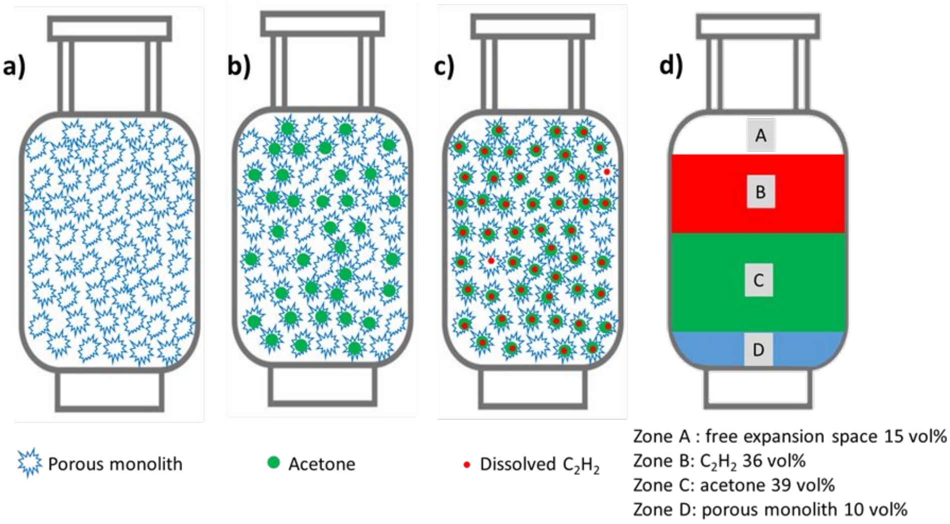

Figure S7. Schematic representation of the C_2H_2 cylinder. a) porous monolith filled, b) acetone filled, and c) C_2H_2 filled. d) Volume percentage of each composition in a charged C_2H_2 cylinder (288 K, 15 bar).

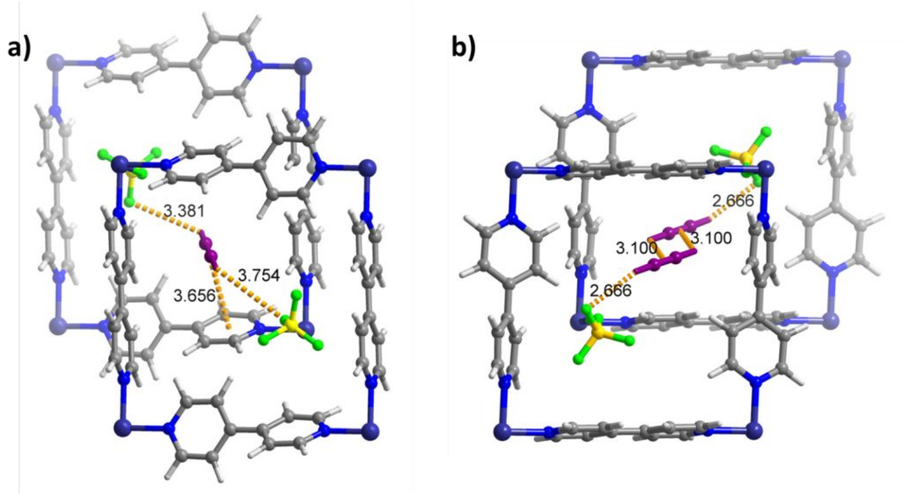

Figure S8. C_2H_2 binding sites in a) **sql-1-Cu-BF$_4$·C_2H_2** and b) **sql-1-Cu-BF$_4$·2C_2H_2** (bond length unit: Å).

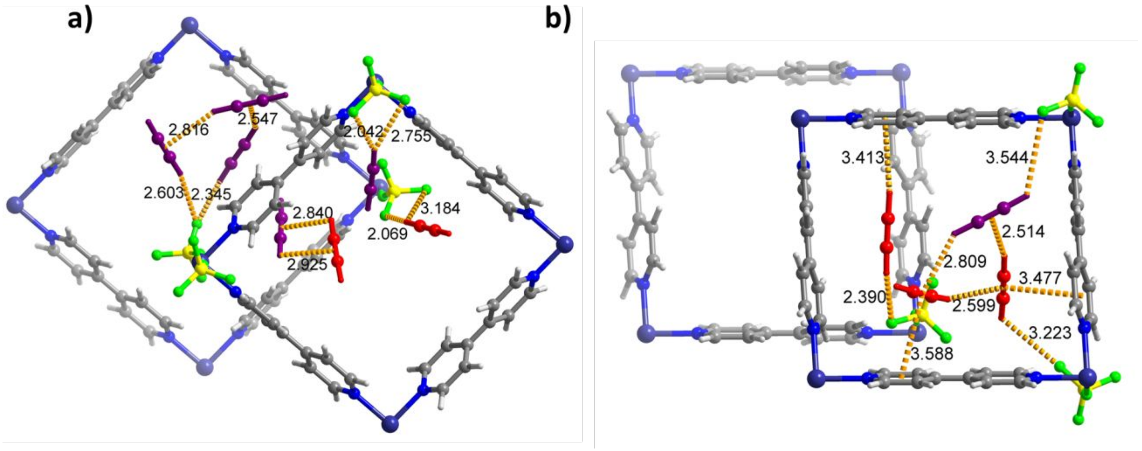

Figure S9. C_2H_2 binding sites in **sql-1-Cu-BF$_4$·6C$_2$H$_2$** (bond length unit: Å).

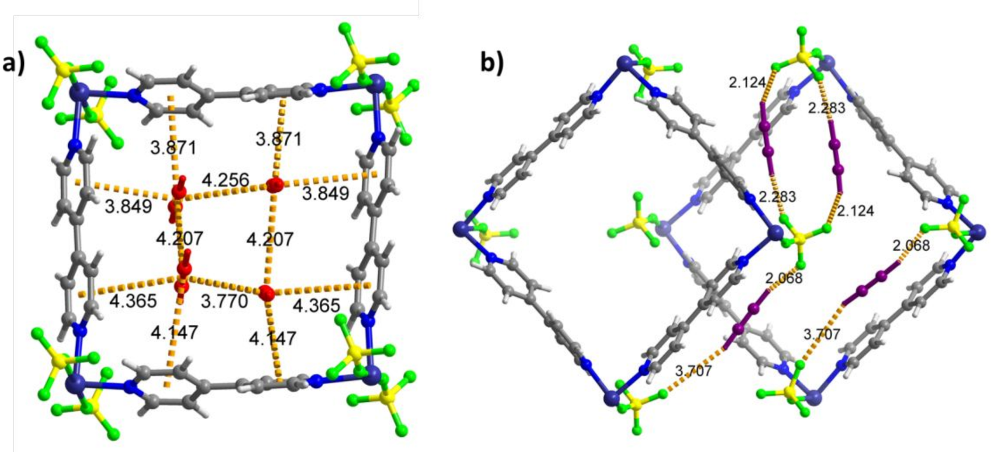

Figure S10. C_2H_2 binding sites in **sql-1-Cu-BF$_4$·8C$_2$H$_2$** (bond length unit: Å).

Table S1. Solubility of acetylene in acetone under different temperatures and pressures. Unit: g/kg (cm^3 g^{-1} STP)

	273 K	278 K	283 K	288 K	293 K	298 K	303 K	313 K	323 K
0 bar	0 (0)	0 (0)	0 (0)	0 (0)	0 (0)	0 (0)	0 (0)	0 (0)	0 (0)
1 bar	58 (50.0)	48.7 (42.0)	41.1 (35.4)	34 (29.3)	27.9 (24.0)	22.4 (19.3)	17.9 (15.4)	10.4 (9.0)	--
2 bar	109.5 (94.3)	95.3 (82.1)	83 (71.5)	72 (62.0)	62.4 (53.8)	53.5 (46.1)	45.7 (39.4)	33 (28.4)	22.7 (19.6)
3 bar	158 (136.1)	137 (118)	122 (105.1)	107.2 (92.4)	94.2 (81.2)	82.2 (70.8)	72.1 (62.1)	54 (46.5)	41.2 (35.5)
5 bar	241 (207.6)	208 (179.2)	182 (156.8)	161 (138.7)	142.3 (122.6)	126.6 (109.1)	113 (97.4)	92.5 (79.7)	75.2 (64.8)
10 bar	526 (453.2)	447 (385.1)	384 (330.8)	335 (288.6)	293 (252.4)	259 (223.1)	230 (198.2)	185 (159.4)	150.5 (129.7)

15 bar	912 (785.7)	754 (649.6)	636 (547.9)	546 (470.4)	472 (406.6)	413 (355.8)	364 (313.6)	289 (249)	234 (201.6)
20 bar	--	1157 (996.8)	958 (825.4)	811 (698.7)	689 (593.6)	597 (514.3)	521 (448.9)	408 (351.5)	327 (281.7)
25 bar	--	--	--	1146 (987.3)	960 (827.1)	822 (708.2)	710 (611.7)	546 (470.4)	432 (372.2)
30 bar	--	--	--	--	1297 (1117.4)	1099 (946.8)	940 (809.8)	709 (610.8)	554 (477.3)

Table S2. Calculation of gravimetric and volumetric working capacity of acetone for C_2H_2 storage/delivery (288 K, 1-15 bar).

Density of liquid C_2H_2	465 g/L (273 K)
Molecular weight of C_2H_2	26 g/mol
Gas molar volume (STP)	22.4 L/mol
Density of liquid acetone	785 g/L (298 K)
Volume percentage of C_2H_2 in the cylinder	36 vol%
Volume percentage of acetone in the cylinder	39 vol%
Mass fraction of C_2H_2 in acetone (288 K, 15 bar)	(465*36)/(785*39) = 54.7 wt% (547 g/kg)
Mass fraction of C_2H_2 in acetone (288 K, 1 bar)	3.4 wt% (34 g/kg)
Gravimetric working capacity of acetone (288 K, 1-15 bar)	54.7-3.4 = 51.3 wt% (513 g/kg)
Volume fraction of liquid C_2H_2 in acetone (288 K, 15 bar)[a]	36/(36+39) = 0.48
Volume fraction of gaseous C_2H_2 (STP) in acetone (288 K, 15 bar)	0.48*465*22.4/26= 192.3 cm^3 cm^{-3}
Volume fraction of gaseous C_2H_2 (STP) in acetone (288 K, 1 bar)	22.5 cm^3 cm^{-3} (34 g/kg)
Volumetric working capacity (STP) of acetone (288 K, 1-15 bar)	192.3-22.5 = 169.8 cm^3 cm^{-3}

a: The expansion of acetone must be taken into account, in this case acetone expanses from 39 vol% to 75 vol%.

Table S3. Summary of sorbents for C_2H_2 sorption at 1 bar and different temperatures.

Materials	C_2H_2 uptake ($cm^3 g^{-1}$)	C_2H_2 uptake ($cm^3 cm^{-3}$)	year	refs
$Cu_2(pzdc)_2(pyz)$	42 (270, 300, 310 K)	74 (270, 300, 310 K)	2005	1
p-tertbutylcalix[4]arene	39 (298 K)	-	2006	2
$Mg(HCOO)_2$	72.5 (196 K); 69.4 (275 K); 65.7 (298 K)	101 (196 K); 96.5 (275 K); 91.3 (298 K)	2007	3
$Mn(HCOO)_2$	68.2 (196 K); 57.7 (275 K); 51.2 (298 K)	112 (196 K); 95.2 (275 K); 84.5 (298 K)		
1-$Cu_2(bpz)$	57.9 (298 K)	79 (298 K)	2008	4
1-$Ag_2(bpz)$	44.1 (298 K)	70.3 (298 K)		
CB[6]	91 (196 K); 52 (298 K)	122 (196 K); 70 (298 K);	2008	5
$[Cu_2(bdc)_2(dabco)]_n$	354.2 (195 K, 0.7 bar); 135 (273 K)<br>60 (298 K); 28 (323 K)	290.4 (195 K, 0.7 bar); 110.7 (273 K)<br>49.2 (298 K); 23 (323 K)	2008	6
$[Cu_2(ndc)_2(dabco)]_n$	176.3 (195 K, 0.7 bar); 136 (273 K)<br>97 (298 K); 56 (323 K)	171 (195 K, 0.7 bar); 132 (273 K)<br>94 (298 K); 54 (323 K)		
$[Cu_2(adc)_2(dabco)]_n$	134 (195 K, 0.7 bar); 96 (273 K)<br>82 (298 K); 62 (323 K)	153 (195 K, 0.7 bar); 109 (273 K)<br>93 (298 K); 71 (323 K)		
$[Zn_2(bdc)_2(dabco)]_n$	345.2 (195 K, 0.7 bar); 203 (273 K)<br>93 (298 K); 44 (323 K)	286.5 (195 K, 0.7 bar); 168.5 (273 K)<br>77 (298 K); 36.5 (323 K)		
$[Zn_2(ndc)_2(dabco)]_n$	191 (195 K, 0.7 bar); 143 (273 K)<br>106 (298 K); 75 (323 K)	185 (195 K, 0.7 bar); 139 (273 K)<br>103 (298 K); 73 (323 K)		
$[Zn_2(adc)_2(dabco)]_n$	132.6 (195 K, 0.7 bar); 112 (273 K)<br>101 (298 K); 84 (323 K)	152.5 (195 K, 0.7 bar); 129 (273 K)<br>116 (298 K); 97 (323 K)		
HKUST-1	201 (295 K); 249 (273 K)	177 (295 K)	2009	7
MOF-505	148 (295 K); 177 (273 K)	137 (295 K)		
MOF-508	90 (290 K)	112 (295 K)		
MIL-53	72 (295 K); 91 (273 K)	67 (295 K)		
MOF-5	26 (295 K); 47 (273 K)	15 (295 K)		
ZIF-8	25 (295 K); 42 (273 K)	23 (295 K)		
$[Cu_2(EBTC)]$	252 (273 K); 160 (295 K)	181 (273 K); 115 (295 K);	2009	8
MAF-2	119 (195 K); 107 (273 K); 70 (298 K)	82 (298 K)	2009	9
$[Co_2(DHTP)]$	197 (295 K)	230 (295 K)	2010	10
$[Mn_2(DHTP)]$	168 (295 K)	182 (295 K)		
$[Mg_2(DHTP)]$	184 (295 K)	167 (295 K)		
$[Zn_2(DHTP)]$	122 (295 K)	150 (295 K)		
$Zn_5(BTA)_6(TDA)_2$	44 (295 K)	57.9 (295 K)	2010	11
$[Zn_4(OH)_2(1,2,4\text{-}BTC)_2]$	53 (295 K)	77.4 (295 K)	2010	12
Cu(BDC-OH)	43 (296 K)	39.1 (296 K)	2010	13
SOF-1a	124 (195 K); 104 (210 K) 87 (230 K);<br>72 (250 K); 61 (270 K)	115 (195 K); 97 (210 K) 81 (230 K);<br>67 (250 K); 57 (270 K);	2010	14
HOF-1a	63 (273 K); 55 (296 K)	53 (273 K); 46 (296 K)	2011	15
UTSA-36a	80 (273 K); 57 (298 K)	80.7 (273 K); 57.5 (298 K)	2011	16
UTSA-38a	64 (295 K)	62 (295 K)	2011	17
UTSA-15a	34 (296 K)	29.4 (296 K)	2011	18

M′MOF-**2a**	195 (195 K); 48 (273 K); 43 (295 K)	194 (195 K); 48 (273 K); 43 (295 K)	2011	19
M′MOF-**3a**	145 (195 K); 45 (273 K); 42 (295 K)	148 (195 K); 46 (273 K); 43 (295 K)		
Yb(bpt)	24 (296 K)	24 (296 K)	2011	20
Cu_4L	193 (273 K); 154 (298 K)	160 (273 K); 128 (298 K)	2012	21
Fe_2(dobdc)	156 (318 K)	176 (318 K)	2012	22
UMCM-150	129 (296 K)	82 (296 K)	2012	23
PCN-16	176 (296 K)	126 (296 K)		
NOTT-101	184 (296 K)	126 (296 K)		
NOTT-102	146 (296 K)	86 (296 K)		
UTSA-20	150 (296 K)	136 (296 K)		
UTSA-33a	84 (296 K); 111 (273 K)	83 (296 K); 110 (273 K)	2012	24
UTSA-34b	121 (296 K)	102 (296 K)	2012	25
UTSA-35a	65 (296 K)	68 (296 K)	2012	26
UTSA-30a	52 (296 K)	56 (296 K)	2012	27
UTSA-5a	59.8 (296 K); 80.2 (273 K)	88 (296 K); 118 (273 K)	2013	
UTSA-48a	40 (296 K); 47 (273 K)	32 (296 K); 37 (273 K)	2013	
UTSA-50a	113.9 (273 K); 90.6 (296 K)	123 (273 K); 98 (296 K)	2013	28
ZJU-5a	290 (273 K); 193 (298 K)	197 (273 K); 131 (298 K)	2013	29
ZJU-26a	127 (273 K); 84 (298 K)	82.5 (273 K); 55 (298 K)	2013	30
ZJU-7a	245 (273 K); 180 (298 K)	135 (298 K)	2014	31
ZJU-60a	178.7 (273 K); 150.6 (296 K)	119 (273 K); 100 (296 K)	2014	32
ZJU-61a	164 (273 K); 139 (298 K)	106 (273 K); 90 (298 K)	2014	33
MIL-101b	118 (313 K)	53 (313 K)	2014	34
MIL-101c	143 (313 K)	64 (313 K)		
Cu–TDPAH	155 (298 K)	120 (298 K)	2014	35
Cu–TDPAT	248.3 (273 K); 177.7 (298 K)	194 (273 K); 139 (298 K)	2014	36
NOTT-300	142 (293 K)	162 (293 K) ρ=1.14 g/cc	2015	37
Cu_2TPTC-Me	257 (273 K); 203 (298 K)	198 (273 K); 156 (298 K)	2015	38
Cu_2TPTC-OMe	248 (273 K); 204 (298 K)	191 (273 K); 157 (298 K)		
UTSA-60a	70 (296 K)	53 (296 K) ρ=0.76 g/cc	2015	39
UTSA-100a	119 (273 K); 95.6 (296 K)	136 (273 K); 110 (296 K)	2015	40
FJI-H8	277 (273 K); 224 (295 K); 206 (303 K); 200 (308 K)	242 (273 K); 196 (295 K) 180 (303 K); 175 (308 K)	2015	41
ZJU-8a	272 (273 K); 195 (298 K);	201 (273 K); 144 (298 K);	2015	42
ZJU-9a	245 (273 K); 193 (298 K);	190 (273 K); 150 (298 K);	2015	43
ZJU-70a	235 (273 K); 191 (298 K)	206 (273 K); 167 (298 K)	2015	44
HOF-3a	58 (273 K); 47 (296 K)	25 (273 K); 20 (296 K)	2015	45
HOF-5a	182 (273 K); 102 (296 K)	174 (273 K); 97 (296 K)	2015	46
ZJU-10a	258 (273 K); 174 (298 K)	195 (273 K); 132 (298 K)	2016	47
ZJNU-46a	257 (278 K); 215 (288 K); 187 (295 K); 176 (298 K); 135 (308 K)	188 (278 K); 158 (288 K); 137 (295 K); 129 (298 K); 99 (308 K)	2016	48
ZJNU-47a	283 (278 K); 240 (288 K) 213 (295 K); 200 (298 K); 167 (308 K)	208 (278 K); 176 (288 K) 157 (295 K); 147 (298 K); 123 (308 K)		

ZJNU-48a	265 (278 K); 220 (288 K); 193 (295 K); 180 (298 K); 146 (308 K)	195 (278 K); 162 (288 K); 142 (295 K); 133 (298 K); 108 (308 K)		
ZJNU-54a	170 (308 K); 200 (298 K); 211 (295 K); 235 (288 K); 259 (278 K)	138 (308 K); 163 (298 K); 172 (295 K); 191 (288 K); 211 (278 K)	2016	49
[Mn(bdc)(dpe)]	86.2 (195 K); 83 (220 K); 74 (240 K); 63 (246 K, 0.85 bar); 9 (268 K); 7 (273 K)	116 (195 K); 111 (220 K); 99 (240 K); 85 (246 K, 0.85 bar); 12 (268 K); 9 (273 K)	2016	50
ZJU-40a	287 (273 K); 216 (298 K)	215 (273 K); 162 (298 K)	2016	51
NJU-Bai17	222.4 (296 K); 295 (273 K)	176 (296 K); 233 (273 K)	2016	52
UTSA-68a	137 (273 K); 116 (298 K)	132 (273 K); 112 (298 K);	2016	
UTSA-74a	128 (273 K); 108 (298 K)	128 (273 K); 172 (298 K);	2016	53
FJU-21a	61 (296 K)	67 (296 K)	2016	54
FJU-22a	114.8 (296 K)	124 (296 K)		
MFM-202a	409.9 (195 K); 146.7 (273 K) 106 (283 K); 77.1 (293 K); 57.8 (303 K)	259 (195 K); 93 (273 K) 67 (283 K); 49 (293 K); 36 (303 K)	2016	55
CPM-231	255.1 (273 K); 177.6 (298 K)	235 (273 K); 164 (298 K)	2016	56
SIFSIX-1-Cu	206 (283 K); 197 (293 K); 195 (296 K); 190 (298 K); 188 (303 K); 177 (313 K)	178 (283 K); 170 (293 K); 168 (296 K); 164 (298 K); 162 (303 K); 153 (313 K)	2016	57
SIFSIX-2-Cu	166 (283 K); 121 (296 K); 121 (298 K); 109 (303 K)	105 (283 K); 77 (296 K); 77 (298 K); 69 (303 K)		
SIFSIX-2-Cu-i	99 (283 K); 90 (298 K); 83 (313 K)	123 (283 K); 112 (298 K); 104 (313 K)		
SIFSIX-3-Ni	81 (273 K); 74 (298 K)	130 (273 K); 119 (298 K)		
SIFSIX-3-Cu	83 (273 K); 78 (298 K)	133 (273 K); 125 (298 K)		
SIFSIX-3-Zn	84 (283 K); 82 (298 K)	132 (283 K); 129 (298 K)		
SIFSIX-14-Cu-i (UTSA-200a)	82 (298 K)	116 (298 K)	2017	
UTSA-90a	214 (295 K); 255 (273 K)	162 (295 K); 193 (273 K)	2017	
UTSA-222a	85.3 (296 K); 103.4 (273 K)	111 (298 K); 135 (273 K)	2017	
UTSA-300a	70 (298 K); 76.4 (273 K)	91 (298 K); 99 (273 K)	2017	
P5-SOF	36 (273 K); 31 (298 K)	-	2017	58
P6-SOF	26 (273 K); 18 (298 K)	-		
[$Co_2(HCOO)_2(CPT)_2$]	178 (273 K); 145 (298 K)	179 (273 K); 146 (298 K)	2017	59
MFM-188a	297 (273 K); 232 (295 K)	213 (273 K); 166.7 (295 K)	2017	60
ZJU-12a	301 (273 K); 244 (298 K)	241 (273 K); 195 (298 K)	2017	61
[$Co_5(OH)(ina)_3(H_2O)_2(TZB)_3$]	135 (273 K); 86 (298 K)	112 (273 K); 71 (298 K)	2017	62
[$Co_3(OH)(tpt)(TZB)_3$]	242 (273 K); 165 (298 K)	194 (273 K); 132 (298 K)		
ZJNU-34(NH_2)	193.8 (298 K); 232.8 (288 K); 269 (278 K)	136.9 (298 K); 164.4 (288 K); 190 (278 K)	2017	63
ZJNU-35(CH_3)	179.8 (298 K); 216.5 (288 K); 258 (278 K)	125.9 (298 K); 151.6 (288 K); 181 (278 K);		
ZJNU-36(NO_2)	175.8 (298 K); 215 (288 K); 254 (278 K);	130.8 (298 K); 159.8 (288 K); 189.3 (278 K)		
ZJNU-37(F)	171.5 (298 K); 209.3 (288 K); 252 (278 K)	120.3 (298 K); 146.8 (288 K); 176.9 (278 K)		
ZJNU-38(CF_3)	153.7 (298 K); 185.1 (288 K); 219 (278 K)	118.6 (298 K); 142.9 (288 K); 169.0 (278 K)		
ZJNU-71	200.4 (298 K); 208.1 (295 K); 222 (288 K); 245 (278 K)	162 (298 K); 168 (295 K); 180 (288 K); 195 (278 K)	2017	64
ZJNU-72	188.2 (298 K); 220 (288 K); 255 (278 K)	141 (298 K); 165 (288 K); 191 (278 K)		
ZJNU-73	189.2 (298 K); 220 (288 K); 255 (278 K)	138 (298 K); 161 (288 K); 186 (278 K)		

ZJNU-74	180 (298 K); 213 (288 K); 245 (278 K)	134 (298 K); 158 (288 K); 182 (278 K)		
NOTT-103	172 (298 K); 213 (288 K); 260 (278 K)	111 (298 K); 137 (288 K); 167 (278 K)		
ZJNU-93a	164 (298 K); 191.1 (288 K); 218.0 (278 K)	134 (298 K); 156 (288 K); 177 (278 K)	2018	65
JCM-1	75 (298 K); 93 (273 K)	100 (298 K); 124 (273 K)	2018	66
FJU-89a	123 (273 K); 101 (296 K)	68 (273 K); 56 (296 K)	2018	67
NKMOF-1-Ni	61 (298 K)	109 (298 K)	2018	68
FJU-36a	66.5 (273 K); 52.2 (296 K)	64 (273 K); 50 (296 K)	2018	69
ZJU-196a	83.5 (298 K); 87 (273 K)	109 (298 K); 113 (273 K)	2018	70
MFM-102	150 (298 K); 251 (273 K)	88 (298 K); 147 (273 K)	2018	71
MFM-102-NO_2	192 (298 K); 292 (273 K)	136 (298 K); 207 (273 K)		
MFM-102-NH_2	158 (298 K); 261 (273 K)	103 (298 K); 170 (273 K)		
MFM-111	131 (298 K); 241 (273 K)	81 (298 K); 149 (273 K)		
MFM-300 (V^{III})	114.2 (323 K); 154.6 (303 K); 181 (273 K)	127 (323 K); 172 (303 K); 201 (273 K)	2018	72
MFM-300 (V^{IV})	88.5 (323 K); 136.6 (303 K); 175 (273 K)	100 (323 K); 155 (303 K); 198 (273 K)		
PCP-31	68 (273 K); 51 (298 K)	-	2018	73
PCP-32	130 (273 K); 84 (298 K)	-		
UPC-100-In	120.2 (298 K)	108 (298 K)	2018	74
UPC-101-Al	132.1 (298 K)	106 (298 K)		
UPC-102-Zr	70.6 (298 K)	34 (298 K)		
$[Co_3(5\text{-Bipa})_2(5\text{-BipaH})_2]$	250 (273 K); 178 (298 K)	292 (273 K); 208 (298 K)	2018	75
SNNU-65-Cu-Ga	228.7 (273 K)	156 (273 K)	2018	76
SNNU-65-Cu-Fe	269.7 (273 K)	-		
SNNU-65-Cu-In	251.2 (273 K)	-		
SNNU-65-Cu-Sc	287.2 (273 K)	-		
SNNU-45	201 (263 K); 193 (273 K); 151 (283 K); 134 (298 K); 108 (308 K)	170 (263 K); 163 (273 K); 127 (283 K); 113 (298 K); 91 (308 K)	2019	77
JNU-1	72 (273 K); 64 (298 K)	107 (273 K); 95 (298 K)	2019	78
FJU-90a	216 (273 K); 180 (298 K)	176 (273 K); 147 (298 K)	2019	79
JXNU-5	70.3 (273 K); 55.9 (298 K)	91 (273 K); 73 (298 K)	2019	80
NbU-3-Mn/Cr	70 (273 K)	-	2019	81
NbU-3-Mn/Fe	68 (273 K)	-		
NbU-3-Mn/V	52 (273 K)	-		
NbU-3-Mn	40 (273 K)	32 (273 K)		
UPC-105	177.5 (273 K); 118.7 (298 K)	107 (273 K); 72 (298 K)	2019	82
UPC-106	178.4 (273 K); 114.7 (298 K)	111 (273 K); 72 (298 K)		
UPC-107	167.3 (273 K); 98.4 (298 K)	107 (273 K); 63 (298 K)		
UPC-108	161.9 (273 K); 96.6 (298 K)	106 (273 K); 63 (298 K)		
UPC-109	133.6 (273 K); 83.5 (298 K)	94 (273 K); 59 (298 K)		
UPC-110	131.7 (273 K); 73.4 (298 K)	92 (273 K); 51 (298 K)		
UPC-111	135.5 (273 K); 88.1 (298 K)	93 (273 K); 60 (298 K)		
UPC-112	130.1 (273 K); 74.4 (298 K)	98 (273 K); 56 (298 K)		
FJI-H23	258.7 (273 K); 162 (298 K)	147.7 (273 K); 92.5 (298 K)	2019	83

Table S4. Observed (obs.) and calculated (cal.) switching pressures at different temperatures.

T/K	$P_{ga}1$/kPa (obs.)	$P_{ga}1$/kPa (cal.)	Deviation/%	$P_{gd}1$/kPa (obs.)	$P_{gd}1$/kPa (cal.)	Deviation/%
273	2.3	2.31	0.43	1.95	1.97	1.0
278	3.0	2.97	1.0	2.55	2.53	0.78
283	3.8	3.79	0.26	3.25	3.22	0.92
288	4.8	4.79	0.21	4.1	4.07	0.73
293	6.0	6.01	0.17	5.1	5.10	0
298	7.5	7.48	0.26	6.3	6.34	0.63
**T/K**	**$P_{ga}2$/kPa (obs.)**	**$P_{ga}2$/kPa (cal.)**	**Deviation/%**	**$P_{gd}2$/kPa (obs.)**	**$P_{gd}2$/kPa (cal.)**	**Deviation/%**
273	19.3	19.43	0.67	16.5	16.51	0.06
278	25.0	25.02	0.08	21.3	21.24	0.28
283	32.0	31.93	0.22	27.0	27.08	0.30
288	40.5	40.41	0.22	34.0	34.23	0.68
293	50.8	50.73	0.14	43.0	42.93	0.16
298	62.7	63.21	0.81	53.5	53.44	0.11
**T/K**	**$P_{ga}3$/kPa (obs.)**	**$P_{ga}3$/kPa (cal.)**	**Deviation/%**	**$P_{gd}3$/kPa (obs.)**	**$P_{gd}3$/kPa (cal.)**	**Deviation/%**
200	9.9	9.85	0.51	3.9	3.90	0
205	14.0	14.00	0	5.6	5.60	0
210	19.5	19.58	0.41	7.9	7.88	0.25
215	27.0	26.95	0.19	10.9	10.93	0.28
220	36.5	36.57	0.19	14.9	14.93	0.20
225	49.0	48.95	0.10	20.2	20.11	0.45
230	65.0	64.70	0.46	26.7	26.75	0.19

Table S5. Crystallographic data of **sql-1-Cu-BF_4** and its gas loaded phases.

	[a]**sql-1-Cu-BF_4**	[a]**sql-1-Cu-BF_4·2CO_2**	[b]**sql-1-Cu-BF_4·3CO_2**	[b]**sql-1-Cu-BF_4·6CO_2**
Formula	$CuC_{20}N_4H_{16}B_2F_8$	$CuC_{20}N_4H_{16}B_2F_8·2CO_2$	$CuC_{20}N_4H_{16}B_2F_8·3CO_2$	$CuC_{20}N_4H_{16}B_2F_8·6CO_2$
Formula weight	549.52	637.54	681.55	813.58
Temperature/K	273	298	195	195
Crystal system	Monoclinic	Monoclinic	Monoclinic	Triclinic
Space group	*C2/c*	*C2/c*	*P2/c*	*P1*
a/Å	12.4227(8)	13.7219(9)	13.6866(4)	11.0894(7)
b/Å	11.1618(6)	11.0542(6)	11.0884(2)	11.1193(5)
c/Å	16.1420(11)	18.7532(10)	18.4649(5)	14.3930(9)
α/°	90	90	90	86.608(6)
β/°	100.534(4)	95.924(3)	94.172(2)	75.513(5)
γ/°	90	90	90	86.791(9)
Volume/Å^3	2200.5(3)	2829.4(3)	2794.8(1)	1713.7(2)
Z	4	4	4	2
ρ_{calc} g/cm^3	1.66	1.50	1.62	1.58
ρ_{net} g/cm^3	1.66	1.29	1.31	1.07
	**sql-1-Cu-BF_4·C_2H_2**	**sql-1-Cu-BF_4·2C_2H_2**	**sql-1-Cu-BF_4·6C_2H_2**	**sql-1-Cu-BF_4·8C_2H_2**
Formula	$CuC_{20}N_4H_{16}B_2F_8·1C_2H_2$	$CuC_{20}N_4H_{16}B_2F_8·2C_2H_2$	$CuC_{20}N_4H_{16}B_2F_8·6C_2H_2$	$CuC_{20}N_4H_{16}B_2F_8·8C_2H_2$
Formula weight	575.58	601.58	705.58	757.58
Crystal system	Triclinic	Triclinic	Triclinic	Triclinic
Space group	*P1*	*P1*	*P1*	*P1*
a/Å	16.7328	13.7219	11.0894	20.5706
b/Å	11.2565	11.0542	11.1193	11.2321
c/Å	15.3213	18.7532	14.393	18.4221
α/°	89.9804	90	86.6080	90
β/°	91.7456	95.924	75.513	110.2896
γ/°	89.9813	90	86.791	90
Volume/Å^3	2884.47	2829.38	1713.75	3992.34
Z	4	4	2	2
ρ_{calc} g/cm^3	1.33	1.42	1.37	1.27
ρ_{net} g/cm^3	1.27	1.29	1.07	0.92

a: Crystallographic data obtained from reference S84; *b*: Crystallographic data obtained from reference S85.

References

1. Matsuda, R.; Kitaura, R.; Kitagawa, S.; Kubota, Y.; Belosludov, R. V.; Kobayashi, T. C.; Sakamoto, H.; Chiba, T.; Takata, M.; Kawazoe, Y.; Mita, Y., Highly controlled acetylene accommodation in a metal-organic microporous material. *Nature* **2005,** *436* (7048), 238-241.
2. Thallapally, P. K.; Dobrzanska, L.; Gingrich, T. R.; Wirsig, T. B.; Barbour, L. J.; Atwood, J. L., Acetylene absorption and binding in a nonporous crystal lattice. *Angew. Chem. Int. Ed.* **2006,** *45* (39), 6506-6509.
3. Samsonenko , D. G.; Kim, H.; Sun, Y.; Kim, G.-H.; Lee, H.-S.; Kim, K., Microporous Magnesium and Manganese Formates for Acetylene Storage and Separation. *Chem. – Asian J.* **2007**, 2 (4), 484-488.
4. Zhang, J.-P.; Kitagawa, S., Supramolecular isomerism, framework flexibility, unsaturated metal center, and porous property of Ag (I)/Cu (I) 3, 3, 5, 5-tetrametyl-4, 4-bipyrazolate. *J. Am. Chem. Soc.* **2008,** *130* (3), 907-917.
5. Lim, S.; Kim, H.; Selvapalam, N.; Kim, K.-J.; Cho, S. J.; Seo, G.; Kim, K., Cucurbit[6]uril: organic molecular porous material with permanent porosity, exceptional stability, and acetylene sorption properties. *Angew. Chem., Int. Ed.* **2008,** *47* (18), 3352-3355.
6. Tanaka, D.; Higuchi, M.; Horike, S.; Matsuda, R.; Ki-noshita, Y.; Yanai, N.; Kitagawa, S., Storage and Sorption Properties of Acetylene in Jungle-Gym-Like Open Frameworks. *Chem. – Asian J.* **2008**, 3 (8-9), 1343-1349.
7. Xiang, S.; Zhou, W.; Gallegos, J. M.; Liu, Y.; Chen, B., Exceptionally High Acetylene Uptake in a Microporous Metal-Organic Framework with Open Metal Sites. *J. Am. Chem. Soc.* **2009,** *131* (34), 12415-12419.
8. Hu, Y.; Xiang, S.; Zhang, W.; Zhang, Z.; Wang, L.; Bai, J.; Chen, B., A new MOF-505 analog exhibiting high acetylene storage. *Chem. Commun.* **2009,** 48, 7551-7553.
9. Zhang, J.-P.; Chen, X.-M., Optimized acetylene/carbon dioxide sorption in a dynamic porous crystal. *J. Am. Chem. Soc.* **2009,** *131* (15), 5516-5521.
10. Xiang, S.; Zhou, W.; Zhang, Z.; Green, M. A.; Liu, Y.; Chen, B., Open metal sites within isostructural metal–organic frameworks for differential recognition of acetylene and extraordinarily high acetylene storage capacity at room temperature. *Angew. Chem. Int. Ed.* **2010,** *49* (27), 4615-4618.
11. Zhang, Z.; Xiang, S.; Chen, Y.-S.; Ma, S.; Lee, Y.; Phely-Bobin, T.; Chen, B., A Robust Highly Interpenetrated Metal− Organic Framework Constructed from Pentanuclear Clusters for Selective Sorption of Gas Molecules. *Inorg. Chem.* **2010,** *49* (18), 8444-8448.
12. Zhang, Z.; Xiang, S.; Rao, X.; Zheng, Q.; Fronczek, F. R.; Qian, G.; Chen, B., A rod packing microporous metal–organic framework with open metal sites for selective guest sorption and sensing of nitrobenzene. *Chem. Commun.* **2010,** *46* (38), 7205-7207.
13. Chen, Z.; Xiang, S.; Arman, H. D.; Li, P.; Tidrow, S.; Zhao, D.; Chen, B., A Microporous Metal–Organic Framework with Immobilized –OH Functional Groups within the Pore Surfaces for Selective Gas Sorption. *Eur. J. Inorg. Chem.* **2010,** *2010* (24), 3745-3749.

14. Yang, W.; Greenaway, A.; Lin, X.; Matsuda, R.; Blake, A. J.; Wilson, C.; Lewis, W.; Hubberstey, P.; Kitagawa, S.; Champness, N. R.; Schroder, M., Exceptional Thermal Stability in a Supramolecular Organic Framework: Porosity and Gas Storage. *J. Am. Chem. Soc.* **2010,** *132* (41), 14457-14469.

15. He, Y.; Xiang, S.; Chen, B., A microporous hydrogen-bonded organic framework for highly selective C_2H_2/C_2H_4 separation at ambient temperature. *J. Am. Chem. Soc.* **2011,** *133* (37), 14570-14573.

16. Das, M. C.; Xu, H.; Xiang, S.; Zhang, Z.; Arman, H. D.; Qian, G.; Chen, B., A new approach to construct a doubly interpenetrated microporous metal–organic framework of primitive cubic net for highly selective sorption of small hydrocarbon molecules. *Chem. – Eur. J.* **2011,** *17* (28), 7817-7822.

17. Das, M. C.; Xu, H.; Wang, Z.; Srinivas, G.; Zhou, W.; Yue, Y.-F.; Nesterov, V. N.; Qian, G.; Chen, B., A Zn 4 O-containing doubly interpenetrated porous metal–organic framework for photocatalytic decomposition of methyl orange. *Chem. Commun.* **2011,** *47* (42), 11715-11717.

18. Chen, Z.; Xiang, S.; Arman, H. D.; Mondal, J. U.; Li, P.; Zhao, D.; Chen, B., Three-Dimensional Pillar-Layered Copper(II) Metal−Organic Framework with Immobilized Functional OH Groups on Pore Surfaces for Highly Selective CO_2/CH_4 and C_2H_2/CH_4 Gas Sorption at Room Temperature. *Inorg. Chem.* **2011,** *50* (8), 3442-3446.

19. Xiang, S.-C.; Zhang, Z.; Zhao, C.-G.; Hong, K.; Zhao, X.; Ding, D.-R.; Xie, M.-H.; Wu, C.-D.; Das, M. C.; Gill, R., Rationally tuned micropores within enantiopure metal-organic frameworks for highly selective separation of acetylene and ethylene. *Nat. Commun.* **2011,** *2*, 204.

20. Guo, Z.; Xu, H.; Su, S.; Cai, J.; Dang, S.; Xiang, S.; Qian, G.; Zhang, H.; O'Keeffe, M.; Chen, B., A robust near infrared luminescent ytterbium metal–organic framework for sensing of small molecules. *Chem. Commun.* **2011,** *47* (19), 5551-5553.

21. Xue, Y.-S.; He, Y.; Ren, S.-B.; Yue, Y.; Zhou, L.; Li, Y.-Z.; Du, H.-B.; You, X.-Z.; Chen, B., A robust microporous metal–organic framework constructed from a flexible organic linker for acetylene storage at ambient temperature. *J. Mater. Chem.* **2012,** *22* (20), 10195-10199.

22. Bloch, E. D.; Queen, W. L.; Krishna, R.; Zadrozny, J. M.; Brown, C. M.; Long, J. R., Hydrocarbon separations in a metal-organic framework with open iron (II) coordination sites. *science* **2012,** *335* (6076), 1606-1610.

23. He, Y.; Krishna, R.; Chen, B., Metal–organic frameworks with potential for energy-efficient adsorptive separation of light hydrocarbons. *Energy Environ. Sci.* **2012,** *5* (10), 9107-9120.

24. He, Y.; Zhang, Z.; Xiang, S.; Fronczek, F. R.; Krishna, R.; Chen, B., A microporous metal–organic framework for highly selective separation of acetylene, ethylene, and ethane from methane at room temperature. *Chem.–Eur. J.* **2012,** *18* (2), 613-619.

25. He, Y.; Zhang, Z.; Xiang, S.; Wu, H.; Fronczek, F. R.; Zhou, W.; Krishna, R.; O'Keeffe, M.; Chen, B., High separation capacity and selectivity of C2 hydrocarbons over methane within a microporous metal–organic framework at room temperature. *Chem. – Eur. J.* **2012,** *18* (7), 1901-1904.

26. He, Y.; Zhang, Z.; Xiang, S.; Fronczek, F. R.; Krishna, R.; Chen, B., A robust doubly interpenetrated metal–organic framework constructed from a novel aromatic tricarboxylate for highly selective separation of small hydrocarbons. *Chem. Commun.* **2012,** *48* (52), 6493-6495.

27. He, Y.; Xiang, S.; Zhang, Z.; Xiong, S.; Fronczek, F. R.; Krishna, R.; O'Keeffe, M.; Chen, B., A microporous lanthanide-tricarboxylate framework with the potential for purification of natural gas. *Chem. Commun.* **2012,** *48* (88), 10856-10858.

28. Xu, H.; He, Y.; Zhang, Z.; Xiang, S.; Cai, J.; Cui, Y.; Yang, Y.; Qian, G.; Chen, B., A microporous metal–organic framework with both open metal and Lewis basic pyridyl sites for highly selective C_2H_2/CH_4 and C_2H_2/CO_2 gas separation at room temperature. *J. Mater. Chem. A* **2013,** *1* (1), 77-81.

29. Rao, X.; Cai, J.; Yu, J.; He, Y.; Wu, C.; Zhou, W.; Yildirim, T.; Chen, B.; Qian, G., A microporous metal–organic framework with both open metal and Lewis basic pyridyl sites for high C_2H_2 and CH_4 storage at room temperature. *Chem. Commun.* **2013,** *49* (60), 6719-6721.

30. Duan, X.; Cai, J.; Yu, J.; Wu, C.; Cui, Y.; Yang, Y.; Qian, G., Three-dimensional copper (II) metal–organic framework with open metal sites and anthracene nucleus for highly selective C_2H_2/CH_4 and C_2H_2/CO_2 gas separation at room temperature. *Microporous and Mesoporous Mater.* **2013,** *181*, 99-104.

31. Cai, J.; Lin, Y.; Yu, J.; Wu, C.; Chen, L.; Cui, Y.; Yang, Y.; Chen, B.; Qian, G., A NbO type microporous metal–organic framework constructed from a naphthalene derived ligand for CH_4 and C_2H_2 storage at room temperature. *RSC Adv.* **2014,** *4* (90), 49457-49461.

32. Duan, X.; Zhang, Q.; Cai, J.; Yang, Y.; Cui, Y.; He, Y.; Wu, C.; Krishna, R.; Chen, B.; Qian, G., A new metal–organic framework with potential for adsorptive separation of methane from carbon dioxide, acetylene, ethylene, and ethane established by simulated breakthrough experiments. *J. Mater. Chem. A* **2014,** *2* (8), 2628-2633.

33. Duan, X.; Zhang, Q.; Cai, J.; Cui, Y.; Wu, C.; Yang, Y.; Qian, G., A new microporous metal–organic framework with potential for highly selective separation methane from acetylene, ethylene and ethane at room temperature. *Microporous and Mesoporous Mater.* **2014,** *190*, 32-37.

34. Lee, S.-J.; Yoon, J. W.; Seo, Y.-K.; Kim, M.-B.; Lee, S.-K.; Lee, U. H.; Hwang, Y. K.; Bae, Y.-S.; Chang, J.-S., Effect of purification conditions on gas storage and separations in a chromium-based metal-organic framework MIL-101. *Microporous Mesoporous Mater.* **2014,** *193*, 160-165.

35. Liu, K.; Li, B.; Li, Y.; Li, X.; Yang, F.; Zeng, G.; Peng, Y.; Zhang, Z.; Li, G.; Shi, Z., An N-rich metal–organic framework with an rht topology: high CO_2 and C_2 hydrocarbons uptake and selective capture from CH_4. *Chem. Commun.* **2014,** *50* (39), 5031-5033.

36. Liu, K.; Ma, D.; Li, B.; Li, Y.; Yao, K.; Zhang, Z.; Han, Y.; Shi, Z., High storage capacity and separation selectivity for C_2 hydrocarbons over methane in the metal–organic framework Cu–TDPAT. *J. Mater. Chem. A* **2014,** *2* (38), 15823-15828.

37. Yang, S.; Ramirez-Cuesta, A. J.; Newby, R.; Garcia-Sakai, V.; Manuel, P.; Callear, S. K.; Campbell, S. I.; Tang, C. C.; Schröder, M., Supramolecular binding and separation of hydrocarbons within a functionalized porous metal–organic framework. *Nat. Chem.* **2015,** *7* (2), 121.

38. Xia, T.; Cai, J.; Wang, H.; Duan, X.; Cui, Y.; Yang, Y.; Qian, G., Microporous metal-organic frameworks with suitable pore spaces for acetylene storage and purification. *Microporous Mesoporous Mater.* **2015,** *215*, 109-115.

39. Wen, H.-M.; Li, B.; Wang, H.; Wu, C.; Alfooty, K.; Krishna, R.; Chen, B., A microporous metal–organic framework with rare lvt topology for highly selective C_2H_2/C_2H_4 separation at room temperature. *Chem. Commun.* **2015,** *51* (26), 5610-5613.

40. Hu, T.-L.; Wang, H.; Li, B.; Krishna, R.; Wu, H.; Zhou, W.; Zhao, Y.; Han, Y.; Wang, X.; Zhu, W., Yao, Z.; Xiang, S.; Chen, B., Microporous metal–organic framework with dual functionalities for highly efficient removal of acetylene from ethylene/acetylene mixtures. *Nat. Commun.* **2015,** *6*, 7328.

41. Pang, J.; Jiang, F.; Wu, M.; Liu, C.; Su, K.; Lu, W.; Yuan, D.; Hong, M., A porous metal-organic framework with ultrahigh acetylene uptake capacity under ambient conditions. *Nat. Commun.* **2015,** *6*, 7575.

42. Cai, J.; Wang, H.; Wang, H.; Duan, X.; Wang, Z.; Cui, Y.; Yang, Y.; Chen, B.; Qian, G., An amino-decorated NbO-type metal–organic framework for high C_2H_2 storage and selective CO_2 capture. *RSC Adv.* **2015,** *5* (94), 77417-77422.

43. Duan, X.; Wang, H.; Cui, Y.; Yang, Y.; Wang, Z.; Chen, B.; Qian, G., A new NbO type metal–organic framework for high acetylene and methane storage. *RSC Adv.* **2015,** *5* (103), 84446-84450.

44. Duan, X.; Wu, C.; Xiang, S.; Zhou, W.; Yildirim, T.; Cui, Y.; Yang, Y.; Chen, B.; Qian, G., Novel Microporous Metal-Organic Framework Exhibiting High Acetylene and Methane Storage Capacities. *Inorg. Chem.* **2015,** *54* (9), 4377-4381.

45. Li, P.; He, Y.; Zhao, Y.; Weng, L.; Wang, H.; Krishna, R.; Wu, H.; Zhou, W.; O'Keeffe, M.; Han, Y., A rod‐packing microporous hydrogen‐bonded organic framework for highly selective separation of C_2H_2/CO_2 at room temperature. *Angew. Chem. Int. Ed.* **2015,** *54* (2), 574-577.

46. Wang, H.; Li, B.; Wu, H.; Hu, T.-L.; Yao, Z.; Zhou, W.; Xiang, S.; Chen, B., A flexible microporous hydrogen-bonded organic framework for gas sorption and separation. *J. Am. Chem. Soc.* **2015,** *137* (31), 9963-9970.

47. Duan, X.; Wang, H.; Ji, Z.; Cui, Y.; Yang, Y.; Qian, G., A novel metal-organic framework for high storage and separation of acetylene at room temperature. *J. Solid State Chem.* **2016,** *241*, 152-156.

48. Song, C.; Jiao, J.; Lin, Q.; Liu, H.; He, Y., C_2H_2 adsorption in three isostructural metal-organic frameworks: boosting C_2H_2 uptake by rational arrangement of nitrogen sites. *Dalton Trans.* **2016,** *45* (11), 4563-4569.

49. Jiao, J.; Dou, L.; Liu, H.; Chen, F.; Bai, D.; Feng, Y.; Xiong, S.; Chen, D.-L.; He, Y., An aminopyrimidine-functionalized cage-based metal–organic framework exhibiting highly selective adsorption of C_2H_2 and CO_2 over CH_4. *Dalton Trans.* **2016,** *45* (34), 13373-13382.

50. Foo, M. L.; Matsuda, R.; Hijikata, Y.; Krishna, R.; Sato, H.; Horike, S.; Hori, A.; Duan, J.; Sato, Y.; Kubota, Y., An adsorbate discriminatory gate effect in a flexible porous coordination polymer for selective adsorption of CO_2 over C_2H_2. *J. Am. Chem. Soc.* **2016,** *138* (9), 3022-3030.

51. Wen, H.-M.; Wang, H.; Li, B.; Cui, Y.; Wang, H.; Qian, G.; Chen, B., A Microporous Metal-Organic Framework with Lewis Basic Nitrogen Sites for High C_2H_2 Storage and Significantly Enhanced C_2H_2/CO_2 Separation at Ambient Conditions. *Inorg. Chem.* **2016,** *55* (15), 7214-7218.

52. Zhang, M.; Li, B.; Li, Y.; Wang, Q.; Zhang, W.; Chen, B.; Li, S.; Pan, Y.; You, X.; Bai, J., Finely tuning MOFs towards high performance in C_2H_2 storage: synthesis and properties of a new MOF-505 analogue with an inserted amide functional group. *Chem. Commun.* **2016,** *52* (45), 7241-7244.

53. Luo, F.; Yan, C.; Dang, L.; Krishna, R.; Zhou, W.; Wu, H.; Dong, X.; Han, Y.; Hu, T.-L.; O'Keeffe, M.; Wang, L.; Luo, M.; Lin, R.-B.; Chen, B., UTSA-74: A MOF-74 Isomer with Two Accessible Binding Sites per Metal Center for Highly Selective Gas Separation. *J. Am. Chem. Soc.* **2016,** *138* (17), 5678-5684.

54. Yao, Z.; Zhang, Z.; Liu, L.; Li, Z.; Zhou, W.; Zhao, Y.; Han, Y.; Chen, B.; Krishna, R.; Xiang, S., Extraordinary Separation of Acetylene ‐ Containing Mixtures with Microporous Metal ‐ Organic Frameworks with Open O Donor Sites and Tunable Robustness through Control of the Helical Chain Secondary Building Units. *Chem. ‐Eur. J.* **2016,** *22* (16), 5676-5683.

55. Gao, S.; Morris, C. G.; Lu, Z.; Yan, Y.; Godfrey, H. G. W.; Murray, C.; Tang, C. C.; Thomas, K. M.; Yang, S.; Schroder, M., Selective Hysteretic Sorption of Light Hydrocarbons in a Flexible Metal-Organic Framework Material. *Chem. Mater.* **2016,** *28* (7), 2331-2340.

56. Zhai, Q.-G.; Bu, X.; Mao, C.; Zhao, X.; Daemen, L.; Cheng, Y.; Ramirez-Cuesta, A. J.; Feng, P., An ultra-tunable platform for molecular engineering of high-performance crystalline porous materials. *Nat. Commun.* **2016,** *7*, 13645.

57. Cui, X. L.; Chen, K. J.; Xing, H. B.; Yang, Q. W.; Krishna, R.; Bao, Z. B.; Wu, H.; Zhou, W.; Dong, X. L.; Han, Y.; Li, B.; Ren, Q. L.; Zaworotko, M. J.; Chen, B. L., Pore chemistry and size control in hybrid porous materials for acetylene capture from ethylene. *Science* **2016,** *353* (6295), 141-144.

58. Tan, L.-L.; Zhu, Y.; Long, H.; Jin, Y.; Zhang, W.; Yang, Y.-W., Pillar[n]arene-based supramolecular organic frameworks with high hydrocarbon storage and selectivity. *Chem. Commun.* **2017,** *53* (48), 6409-6412.

59. Chen, D.-M.; Liu, X.-H.; Tian, J.-Y.; Zhang, J.-H.; Liu, C.-S.; Du, M., Microporous cobalt(II)-organic framework with open O-donor sites for C_2H_2 storage and C_2H_2/CO_2 separation at room temperature. *Inorg. Chem.* **2017,** *56* (24), 14767-14770.

60. Moreau, F.; da Silva, I.; Al Smail, N. H.; Easun, T. L.; Savage, M.; Godfrey, H. G. W.; Parker, S. F.; Manuel, P.; Yang, S.; Schröder, M., Unravelling exceptional acetylene and carbon dioxide adsorption within a tetra-amide functionalized metal-organic framework. *Nat. Commun.* **2017,** *8*, 14085.

61. Duan, X.; Cui, Y.; Yang, Y.; Qian, G., A novel methoxy-decorated metal-organic framework exhibiting high acetylene and carbon dioxide storage capacities. *Crystengcomm* **2017,** *19* (11), 1464-1469.

62. Chen, D.-M.; Zhang, N.-N.; Tian, J.-Y.; Liu, C.-S.; Du, M., Pore modulation of metal–organic frameworks towards enhanced hydrothermal stability and acetylene uptake via incorporation of different functional brackets. *J. Mater. Chem. A* **2017,** *5* (10), 4861-4867.

63. Chen, F.; Bai, D.; Wang, X.; He, Y., A comparative study of the effect of functional groups on C_2H_2 adsorption in NbO-type metal–organic frameworks. *Inorg. Chem. Front.* **2017,** *4* (6), 960-967.

64. Chen, F.; Bai, D.; Jiang, D.; Wang, Y.; He, Y., A comparative study of C_2H_2 adsorption properties in five isomeric copper-based MOFs based on naphthalene-derived diisophthalates. *Dalton Trans.* **2017,** *46* (34), 11469-11478.

65. Li, S.; Wu, J.; Gao, X.; He, M.; Wang, Y.; Wang, X.; He, Y., A NbO-type MOF based on an aromatic-rich and N-functionalized diisophthalate ligand for high-performance acetylene storage and purification. *Crystengcomm* **2018,** *20* (44), 7178-7183.

66. Lee, J.; Chuah, C. Y.; Kim, J.; Kim, Y.; Ko, N.; Seo, Y.; Kim, K.; Bae, T. H.; Lee, E., Separation of Acetylene from Carbon Dioxide and Ethylene by a Water‐Stable Microporous Metal‐Organic Framework with Aligned Imidazolium Groups inside the Channels. *Angew. Chem. Int. Ed.* **2018,** *57* (26), 7869-7873.

67. Ye, Y.; Chen, S.; Chen, L.; Huang, J.; Ma, Z.; Li, Z.; Yao, Z.; Zhang, J.; Zhang, Z.; Xiang, S., Additive-Induced Supramolecular Isomerism and Enhancement of Robustness in Co(II)-Based MOFs for Efficiently Trapping Acetylene from Acetylene-Containing Mixtures. *ACS Appl. Mater. Interfaces* **2018,** *10* (36), 30912-30918.

68. Peng, Y. L.; Pham, T.; Li, P.; Wang, T.; Chen, Y.; Chen, K. J.; Forrest, K. A.; Space, B.; Cheng, P.; Zaworotko, M. J., Robust ultramicroporous metal–organic frameworks with benchmark affinity for acetylene. *Angew. Chem. Int. Ed.* **2018,** *57* (34), 10971-10975.

69. Liu, L.; Yao, Z.; Ye, Y.; Chen, L.; Lin, Q.; Yang, Y.; Zhang, Z.; Xiang, S., Robustness, Selective Gas Separation, and Nitrobenzene Sensing on Two Isomers of Cadmium Metal–Organic Frameworks Containing Various Metal–O–Metal Chains. *Inorg. Chem.* **2018,** *57* (20), 12961-12968.

70. Zhang, L.; Jiang, K.; Li, L.; Xia, Y.-P.; Hu, T.-L.; Yang, Y.; Cui, Y.; Li, B.; Chen, B.; Qian, G., Efficient separation of C_2H_2 from C_2H_2/CO_2 mixtures in an acid–base resistant metal–organic framework. *Chem. Commun.* **2018,** *54* (38), 4846-4849.

71. Duong, T. D.; Sapchenko, S. A.; da Silva, I.; Godfrey, H. G. W.; Cheng, Y.; Daemen, L. L.; Manuel, P.; Ramirez-Cuesta, A. J.; Yang, S.; Schroder, M., Optimal Binding of Acetylene to a Nitro-Decorated Metal-Organic Framework. *J. Am. Chem. Soc.* **2018,** *140* (47), 16006-16009.

72. Lu, Z.; Godfrey, H. G. W.; da Silva, I.; Cheng, Y.; Savage, M.; Manuel, P.; Rudic, S.; Ramirez-Cuesta, A. J.; Yang, S.; Schroder, M., Direct observation of supramolecular binding of light hydrocarbons in vanadium(III) and (IV) metal-organic framework materials. *Chem. Sci.* **2018,** *9* (13), 3401-3408.

73. Lyu, H.; Zhang, Q.; Wang, Y.; Duan, J., Unified meso-pores and dense Cu^{2+} sites in porous coordination polymers for highly efficient gas storage and separation. *Dalton Trans.* **2018,** *47* (13), 4424-4427.

74. Fan, W.; Wang, X.; Xu, B.; Wang, Y.; Liu, D.; Zhang, M.; Shang, Y.; Dai, F.; Zhang, L.; Sun, D., Amino-functionalized MOFs with high physicochemical stability for efficient gas storage/separation, dye adsorption and catalytic performance. *J. Mater. Chem. A* **2018,** *6* (47), 24486-24495.

75. Guo, Y.-Q.; Chang, T.; Liu, X.-H., A highly porous polyhedron-based metal-organic framework exhibiting large C_2H_2 storage capability. *Inorg. Chem. Commun.* **2018,** *87*, 17-19.

76. Zhang, J.-W.; Hu, M.-C.; Li, S.-N.; Jiang, Y.-C.; Qu, P.; Zhai, Q.-G., Assembly of $[Cu_2(COO)_4]$ and $[M_3(\mu_3\text{-}O)(COO)_6]$ (M = Sc, Fe, Ga, and In) building blocks into porous frameworks towards ultra-high C_2H_2/CO_2 and C_2H_2/CH_4 separation performance. *Chem. Commun.* **2018,** *54* (16), 2012-2015.

77. Li, Y.-P.; Wang, Y.; Xue, Y.-Y.; Li, H.-P.; Zhai, Q.-G.; Li, S.-N.; Jiang, Y.-C.; Hu, M.-C.; Bu, X., Ultramicroporous Building Units as a Path to Bi-microporous Metal-Organic Frameworks with High Acetylene Storage and Separation Performance. *Angew. Chem. Int. Ed.* **2019,** *58* (38), 13590-13595.

78. Zeng, H.; Xie, M.; Huang, Y. L.; Zhao, Y. F.; Xie, X. J.; Bai, J. P.; Wan, M. Y.; Krishna, R.; Lu, W. G.; Li, D., Induced Fit of C_2H_2 in a Flexible MOF Through Cooperative Action of Open Metal Sites. *Angew. Chem. Int. Ed.* **2019,** *58* (25), 8515-8519.

79. Ye, Y.; Ma, Z.; Lin, R.-B.; Krishna, R.; Zhou, W.; Lin, Q.; Zhang, Z.; Xiang, S.; Chen, B., Pore Space Partition within a Metal–Organic Framework for Highly Efficient C_2H_2/CO_2 Separation. *J. Am. Chem. Soc.* **2019,** *141* (9), 4130-4136.

80. Liu, R.; Liu, Q.-Y.; Krishna, R.; Wang, W.; He, C.-T.; Wang, Y.-L., Water-stable Europium 1,3,6,8-Tetrakis(4-carboxylphenyl)pyrene Framework for Efficient C_2H_2/CO_2 Separation. *Inorg. Chem.* **2019,** *58* (8), 5089-5095.

81. Jiang, L.; Wu, N.; Li, Q.; Li, J.; Wu, D.; Li, Y., Heterometallic Strategy for Enhancing the Dynamic Separation of C_2H_2/CO_2: A Linear Pentanuclear Cluster-Based Metal–Organic Framework. *Inorg. Chem.* **2019,** *58* (7), 4080-4084.

82. Fan, W.; Wang, X.; Liu, X.; Xu, B.; Zhang, X.; Wang, W.; Wang, X.; Wang, Y.; Dai, F.; Yuan, D.; Sun, D., Regulating C_2H_2 and CO_2 Storage and Separation through Pore Environment Modification in a Microporous Ni-MOF. *ACS Sustain. Chem. Eng.* **2019,** *7* (2), 2134-2140.

83. Huang, P.; Chen, C.; Hong, Z.; Pang, J.; Wu, M.; Jiang, F.; Hong, M., Azobenzene Decorated NbO-Type Metal-Organic Framework for High-Capacity Storage of Energy Gases. *Inorg. Chem.* **2019,** *58* (18), 11983-11987.

84. Hiraide, S.; Tanaka, H.; Miyahara, M. T., Understanding gate adsorption behaviour of CO_2 on elastic layer-structured metal-organic framework-11. *Dalton Trans.* **2016,** *45* (10), 4193-4202.

85. Hiraide, S.; Tanaka, H.; Ishikawa, N.; Miyahara, M. T., Intrinsic Thermal Management Capabilities of Flexible Metal-Organic Frameworks for Carbon Dioxide Separation and Capture. *ACS Appl. Mater. Interfaces* **2017,** *9* (46), 41066-41077.
